# Effect of Experimental Parameters on the Extraction of Grape Seed Oil Obtained by Low Pressure and Supercritical Fluid Extraction

**DOI:** 10.3390/molecules25071634

**Published:** 2020-04-02

**Authors:** Rita de Cássia de Souza, Bruna Aparecida Souza Machado, Gabriele de Abreu Barreto, Ingrid Lessa Leal, Jeancarlo Pereira dos Anjos, Marcelo Andrés Umsza-Guez

**Affiliations:** 1 Federal University of Bahia, Bahia, Salvador 40170-115, Brazil; rcsouza.eng@gmail.com (R.d.C.d.S.); marcelo.umsza@ufba.br (M.A.U.-G.); 2 University Center SENAI CIMATEC, SENAI Institute of Innovation (ISI) in Advanced Health Systems (CIMATEC ISI SAS), National Service of Industrial Learning–SENAI, Bahia, Salvador 41650-010, Brazil; gabriele.barreto@fieb.org.br (G.d.A.B.); ingrid.leal@fieb.org.br (I.L.L.); jeancarlo.anjos@fieb.org.br (J.P.d.A.)

**Keywords:** *Vitis vinifera*, cold extraction, α-tocopherol, supercritical CO_2_ extraction, agro-industrial waste

## Abstract

Grape seeds are an important byproduct from the grape process. The objective of this work was to evaluate the effect of experimental parameters (temperature and time of pretreatment with ultrasound) to obtain grape seed oil using low pressure (Soxhlet-Sox and Bligh Dyer-BD) and high pressure (supercritical carbon dioxide-SFE) methods. The best condition for pretreatment of samples was 30 min of sonication at 30 °C before extraction by Sox or BD. Ultrasound pretreatment was efficient to increase oil extraction yield by 32.10 (Sox), 20.31 (BD) and 12.54% (SFE), depending on the extraction method used as well as, and certainly influenced the total phenolic concentration in 311 (Sox), 234 (BD), and 184 (SFE)%. Ten fatty acids were identified in the oils, the major ones being 18:2ω-6cis (linoleic 52.39%–63.12%), 16:0 (palmitic 20.22%–26.80%) and 18:0 (stearic 8.52%–13.68%). The highest epicatechin concentration was identified in the BD sample: 30-30 (150.49 ± 5.98mg/kg), which presented a concentration of ≥3 times compared to the control (56.68 ± 1.81mg/kg). Ultrasound pretreatment also contributed positively (56% and 99% increase) in the α-tocopherol content of the SFE: 30-30 and BD: 30-30 samples, respectively. The results indicate that the ultrasound pretreatment is a suitable technology to improve the quality of the oil from the grape seed.

## 1. Introduction

Grapes (**Vitis vinifera* L.*) are one of the most widely grown fruit crops in the world, and are popularly consumed not only in fresh fruit forms, but also as processed products and derivatives, including dried fruits, jellies, juices, and wines, with about 70% of its crop intended especially for wine production [1,2,3,4]. Grape seeds are an important byproduct in the grape processing process, although not commercially explored [5], and if improperly discarded they generate environmental problems due to the large volume produced [6]. However, this by-product can be considered as a valuable source of health-beneficial compounds that could add value to products in the food industries [7,8], cosmetics [9] and pharmaceutical [4,10], and also as a raw material for biofuel production [11,12]. Fernandes et al. [13] reported that grape seeds constitute 38% to 52% of dry grape pomace and yield 10%–15% of oil of considerable nutritional value, rich in unsaturated fatty acids, mainly linoleic and oleic acids [14,15]. In addition, this oil may contain higher α-tocopherol levels than those found in canola and soybean oils [16,17,18], as well as presenting compounds. antioxidants of the phenolic compound class [19,20,21].

It is noteworthy that the quality and composition of the extracts and products obtained from plant samples are fundamentally related to the extraction technique employed [14,22]. Grape seed oil is most commonly extracted by the pressing process or through processes that use organic solvents (Bligh and Dyer or Soxhlet). Cold extraction of oils is often associated with a lower final yield, while solvent extraction, despite the better yield, has as disadvantages a longer process time, presence of toxic residues in the final product and lower nutritional quality of the oil [22]. On the other hand, supercritical fluid extraction (SFE) has attracted considerable attention in recent years as a promising alternative to conventional extraction especially in the food, pharmaceutical, cosmetic, and nutraceutical industries due to the higher quality of the products obtained [17,23], and also because it is considered a clean technology (green process/ecofriendly) [18,22,24]. SFE has advantages over traditional techniques in that it allows the use of low environmental impact solvents, usually using supercritical carbon dioxide (CO_2_) as solvent/extraction fluid, and is ideal for the extraction of thermally sensitive compounds, as low temperatures can be employed in the process. According to Machado et al. [24] when comparing the extracts obtained by SFE with those of other conventional extractive methods, it is observed that the number of compounds obtained by SFE from the same matrix is much higher. However, despite the higher number of extracted compounds, the efficiency of the extraction process is often lower, which may indicate greater selectivity. The application of combined ultrasound to SFE has recently been proposed as a mechanism to intensify the process, increase yield and extraction of compounds of interest [25,26,27]. The use of ultrasound has been recognized for its potential for industrial application through processes that can increase extraction yield, quality of extracted oil, process safety, and reduce extraction time. The efficiency of this technique has been attributed to ultrasound wave propagation and cavitation phenomena [28].

Recently, it was demonstrated that the yield of grape seed oil obtained by ultrasound extraction was similar to that obtained by Soxhlet, however, with a shorter extraction time [20,29]. In the studies conducted by Lin et al. [30], Goula et al. [31] and Moghimi, Farzaneh, and Bakhshabadi [28] have reported that pretreatment of the samples by ultrasound may show improvements in oil yields obtained from hemp, pomegranate and black cumin seeds, respectively. In other studies, the combination of SFE and ultrasound techniques led to a higher yield during passion fruit seed oil extraction [26,32].

Thus, the application of ultrasound technology as a step for pretreatment of the sample prior to the process of obtaining the compounds and/or products of interest can contribute positively to the reduction of the extraction time and, in addition, increase the yield of various compounds and lower production costs to obtain the extracts–compounds–products of interest [25,26,33,34]. Within this context, and based on economic factors (yield, high value-added product, etc.) [35], health benefits (bioactive compounds) [5,14] and environmental issues (use of agro-industrial waste and green technology) [4], the aim of this study was to evaluate the influence of ultrasound application (at different times and temperatures) as a method of pretreatment for oil extraction from grape seeds (var. *Syrah*) using low (Soxhlet-Sox and Bligh and Dyer-BD) and high pressure (SFE) methods to obtain oil. In addition, this study also investigated the effects of pretreatment variables and different extraction methods in relation to oil yield, fatty acid profile, activity and content of antioxidant compounds and thermal characteristics of the obtained oils.

## 2. Results and Discussion

**Figure 1:**
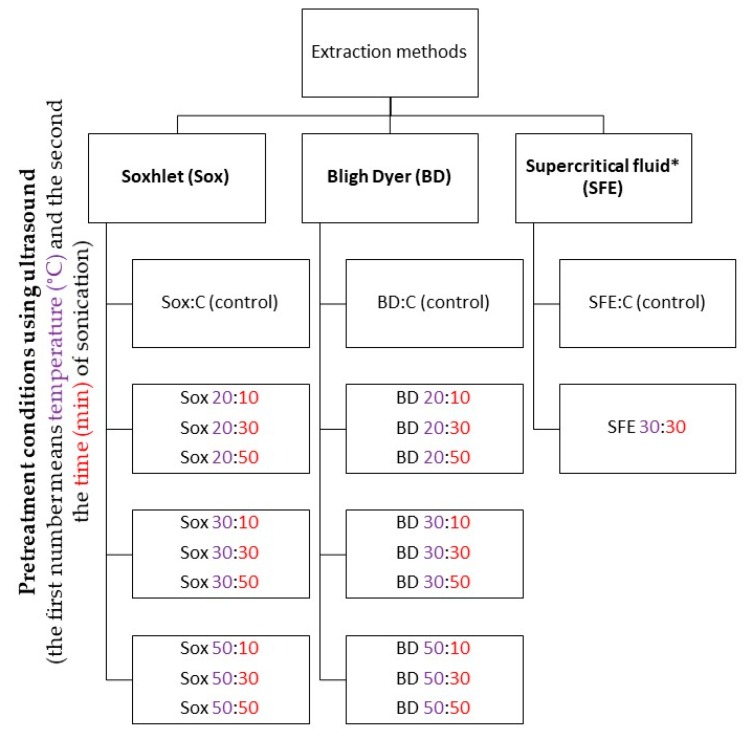
Experimental parameters for obtaining grape seed oil using different treatment conditions (time and temperature) with ultrasound and control sample (CC) obtained by different methods (BD: Bligh and Dyer; Sox: Soxhlet, and supercritical fluid extraction (SFE)). Pretreatment conditions using ultrasound: the first number means temperature (°C) (purple) and the second number means the time (min) (red) of sonication. BD: Bligh and Dyer; Sox: Soxhlet; SFE: Extraction with supercritical fluid; C (control)—corresponding the control samples. * SFE performed after defining the best pretreatment ultrasound condition for conventional techniques (BD and Sox).

Figure 1 shows experimental parameters for the pretreatment of grape seed samples (var. Syrah) with ultrasound (frequency 50–60 Hz) and the three extraction methods investigated for each condition and obtaining controls.

### 2.1. Yield

**Figure 2:**
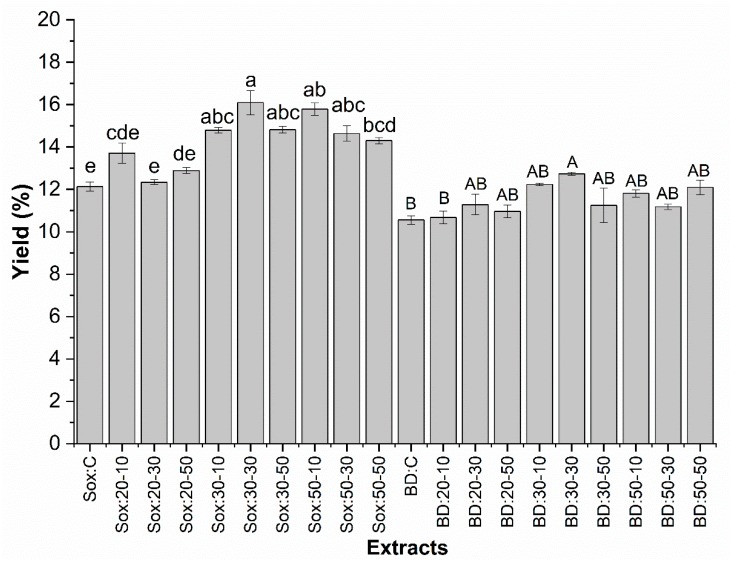
Determination of yield (%) of grape seed oil using different treatment conditions (time and temperature) with ultrasound and control sample (C) obtained by low pressure methods (Sox and BD). The bars in each column represent the standard error. Samples with the same lowercase letter (Sox extraction) and the same uppercase letter (BD extraction), showed no significant difference (p > 0.05) by the Tukey test with 95% confidence.

Figure 2 presents the results for the yield (%) of the oils obtained by the low-pressure extraction methods (Sox = Soxhlet and BD = Bligh and Dyer) under the different conditions employed, as well as the respective controls (without ultrasound treatment). The yield ranged from 12.1 (C) to 16.1% (30-30) for Sox and from 10.5% (C) to 12.7% (30-30) for BD extraction, with significant differences between the samples.

In general, the extraction yield by Sox method was higher than by BD under the conditions evaluated in this study. As shown in Figure 2, for Sox extraction, the condition Sox: 30-30 showed the highest oil yield, with an increase of approximately 33% compared to the control sample (Sox: C). This condition (Sox: 30-30) was significantly different (higher) than the yields at 20 °C and control sample, but with the exception of the Sox: 50-50 sample, there was no significant difference in extractions at 50 °C and 30 °C. Oil yield could be influenced by extraction time and temperature used in the application of the ultrasound technique [36]. There are few studies reporting the application of the ultrasound technique in the extraction of grape seed oil. [20] obtained grape seed oil yield (14.08%) extracted by ultrasound (20 kHz, 150 W, 30 min) similar to that obtained by Sox extraction (14.64%). However, Malicanin et al. [29] obtained in the ultrasound extraction (35 kHz, 150 W, 120 min) of grape seed oil (8.39%) a significantly lower yield (p < 0.05) than that obtained by Sox (11%, 25%). Comparing these literature data obtained by Sox extraction with the best extraction condition of this study (Sox: 30-30), the positive influence of ultrasound pretreatment was observed, with a yield of 9.97% (C) and 43.1% (30:30) higher in grape seed oil, respectively. In addition, in BD: 30-30 (Figure 2) the pretreated seeds showed a 13% increase in oil yield compared to the control sample (BD: C). Araújo et al. [37] observed that pretreatment with ultrasound and subsequent extraction by BD (52.5%) resulted in a higher extraction of microalgae lipids than that obtained by applying the Sox method (1.8%) without treatment. It is noteworthy to observe that, with the exception of samples BD: C and BD: 20–10, the oils obtained under other extraction conditions applied in this study did not present significant differences among themselves in relation to sample BD: 30–30. As mentioned in other studies, ultrasonic technology greatly improves the extraction efficiency of compounds of interest within a given time [38], and coupled with temperature could facilitate the transfer of oil from seed to heated medium due to the increase in the number of cavitation bubbles that will allow a larger matrix-solvent contact area. For oil extraction, this effect may also be due to the fact that the sonication time alters the stability of the oily body that separated the oil from the rest of the cellular content oily body consist of a triglyceride matrix surrounded by a monolayer of phospholipids linked together with proteins called oleosins and caleosin. They completely cover the surface of the oily bodies, stabilizing them. The sonication can promote, by coagulation of oleosins and kerosine, the rupture of oil bodies and facilitate oil extraction [20]. Elevated temperature and sonication time are important variables as they can affect biologically active compounds in the oil that are thermally unstable and/or photosensitive [36].

The highest yield efficiency found for both methods was at 30:30, indicating that this time was sufficient for the cavitation phenomenon promoted by ultrasound application, thus, these conditions were adopted for the extraction of grape seed oil by the supercritical extraction method.

**Figure 3:**
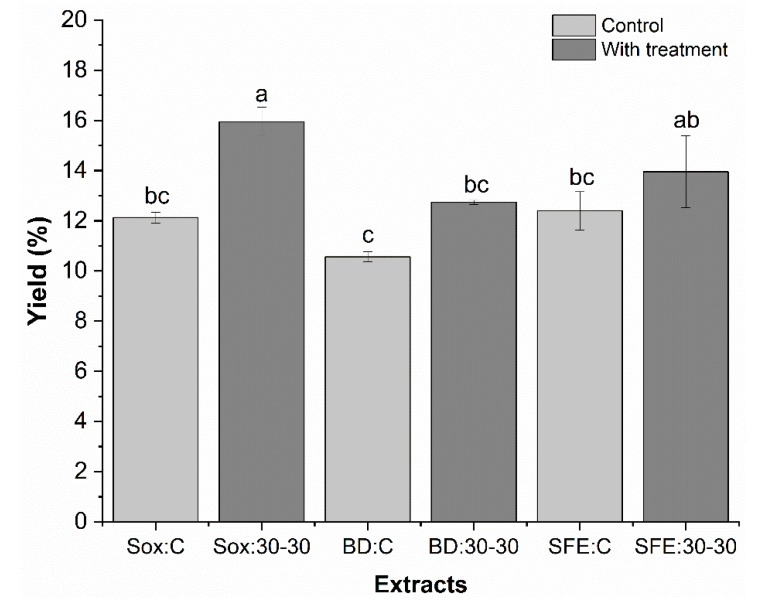
Determination of yield (%) of grape seed oils obtained by low pressure (Sox and BD) and supercritical extraction (SFE) methods. The bars in each column represent the standard error. Samples with the same letter showed no significant difference (p > 0.05) by the Tukey test with 95% confidence.

Figure 3 presents the results for the yield (%) of grape seed oil obtained by three extraction methods (low pressure Sox and BD and high pressure—SFE) at condition 30:30, as well as the respective controls. The oil yield for supercritical extraction was 12.3 (SFE: C) and 13.9% (SFE: 30–30).

Seed ultrasound pretreatment (SFE: 30-30) may have caused an increase in the solvent CO_2_ penetration rate in the tissue, increasing mass transfer compared to control (SFE: C), contributing to an increase in oil yield approximately 12.5%. Similar results were reported by Barrales et al. [26], who obtained 13% yield of ultrasound-assisted SFE-CO_2_ (26 MPa) passion fruit seed oil. The increased yield can be attributed to the high extraction rates achieved in the ultrasound application process as previously mentioned. The combined action of ultrasound and supercritical CO_2_ has been proven to improve the efficiency of extracting different compounds using other matrices: alday oil [39,40], blackbarry antioxidants [25], capsaicinoids from chilli pepper [41], and sweet passion fruit seed oil [32].

Comparing the three extraction methodologies (Figure 3), a positive influence of the ultrasound pretreatment on the grape seeds was observed, noticing that in terms of oil yields, Sox < SFE < BD at condition 30-30, respectively. The condition Sox: 30-30 is statistically different (p < 0.05) from the other conditions evaluated, except for the SFE: 30-30. Better yield achieved by pretreatment Sox and SFE methods can be attributed to longer Sox extraction times and greater SFE selectivity, which may have been improved by ultrasound application [25]. However, SFE has the advantage over Sox extraction by reducing the extraction time by 4.5 h, as well as allowing the use of non-toxic solvents such as CO_2_ and enabling lower temperature extraction, preserving the thermolabile oil compounds [24,32].

### 2.2. Fatty Acid Profile

Table 1 presents the composition and quantification of fatty acids by normalization, the sum of saturated, monounsaturated and polyunsaturated fatty acids of grape seed oils obtained by the low pressure extraction methods (Sox and BD) under different conditions of ultrasound, as well as, the high pressure extraction method (SFE) in the best extraction condition, and the respective controls for the three methods (without ultrasound pretreatment). Seven different fatty acids were identified in all oil samples. The majorities (minimum and maximum quantified in the different extraction conditions) were: 18:2 ω-6 cis (linoleic 52.39%–63.12%) 16:0 (palmitic 18.56%–27.28%), 18:0 (stearic 8.77%–1.65%), 18:1 ω-9 cis (oleic 4.49%–7.48%), and 18:3 ω-3 cis (linolenic 0.13%–0.20%) representing approximately 97% of the total fatty acids present in the samples evaluated

The average (minimum and maximum) found for the sum, saturated, monounsaturated and polyunsaturated values of the samples of the present study ranged from 30.06% to 42.06%, 4.56% to 7.61% and 52.53% to 63, 29% respectively (Table 2).

The results obtained for Sox: 30-30 showed a significant difference (p < 0.05) in the polyunsaturated fatty acid fraction compared to all oil samples obtained in this methodology. For BD, the conditions BD: 30-30, BD: 30-10 and BD: 20-50 were not statistically different in terms of PUFA (polyunsaturated fatty acid) content, but were significantly higher (p < 0.05) than the other extraction conditions. When comparing the 30:30 condition with a control sample (C), it was observed that the seed pretreatment positively influenced the essential fatty acid extraction obtained by Sox, with an increase of 6.69% and 27.33% of linoleic (ω-6) and linolenic (ω-3) acids, respectively. For BD there was 3.97% increase in linoleic acid (ω-6) with pretreatment, but it did not show significant difference for linolenic acid. It is important to notice that pretreatment in 30:30 condition may lead to the extraction of larger amounts of PUFA. However, by increasing the temperature to 50 °C at the same time of sonication, the amount of fatty acid is lower. This may indicate that the adjustment of variables during pretreatment may influence extraction, allowing the solvent to reach the solute-containing matrix spaces more easily, solubilizing a larger amount and increasing the yield of some compounds [42].

As shown in Table 2, it is noticeable that for all extraction methodologies, regardless of ultrasound pretreatment, the major fatty acid was linoleic, followed by palmitic acid as the second largest fatty acid present. Regarding the concentration of linoleic, as the most abundant fatty acid in grape seed oil, the values found in this work (52%–63%) are in agreement with those found in the literature, however, the amount found for palmitic acid (20.45%–27.31%), was much higher (≥3 times) when compared to the following works of grape seed oil: Fernandes et al. [13], Freitas et al. [43]; Da Porto et al. [44] and Coelho et al. [6] who found 60.4%–65.4% and 8.0%–9.0% (Tinta Barroca grape); 62.6%–69.1% and 6.55%–9.81% (Herbemont and Isabel); 71.5%–72.3% and 6.83%–7.22% (Raboso Piave); 64.5%–67.3%; and 7.53%–8.13% (Portuguese) of linoleic and palmitic acid respectively.

No studies were found in the literature about the fatty acid profile of grape oil of Syrah variety grown in Brazil, however in a study conducted by Amorim et al. [8] was found for a Syrah grape peel product from the São Francisco Valley, a palmitic acid concentration of 26.37%, a percentage similar to that shown in Table 3. The variations may be due to different extraction conditions used and the use of different grape varieties. Considering the general classification of fatty acids, it was found that the grape seed oil presented higher value of polyunsaturated fatty acids in all pretreatment conditions and also in the control sample. These data are in accordance with the literature for grape seed oil extracted by Sox [20,45], cold extraction [19,46], and SFE [14].

Table 3 presents the quantification of major fatty acids by normalization, the sum of saturated (SFA), monounsaturated (MUFA), and polyunsaturated (PUFA) fatty acid oils obtained from the three extraction methods in the best process condition (30:30) and its controls. It is observed that the extraction methodology used and the application of ultrasound do not affect the fatty acid profile, but contributed to the increase of 1.80%, 4.05%, and 0.98% of the major fatty acid (acid linoleic) extracted by Sox: 30-30, BD: 30-30, and SFE: 30-30 respectively, when compared with the control sample.

**Figure 4:**
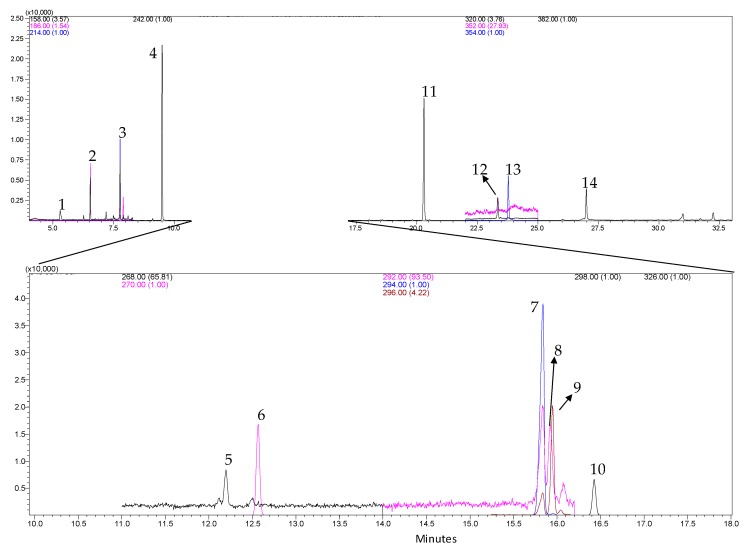
Chromatogram obtained by GC-MS (SIM mode) for the fatty acid profile of grape seed oil using SFE 30:30. Peaks identification: 1—C8:0 (Methyl Octanoate, 5.32 min); 2—C10:0 (Methyl Decanoate, 6.54 min); 3—C12:0 (Methyl Laurate, 7.76 min); 4—C14:0 (Methyl Tetradecanoate, 9.51 min); 5—C16:1 (Methyl Palmitoleate, 12.21 min) 6—C16:0 (Methyl Palmitate, 12.5 min); 7—C18:3 (Methyl Linoleate, 15.8 min); 8—C18:3 (Methyl Linolenate, 15.91 min); 9—C18:1 (cis-9-Oleic Methyl Ester, 15.93 min); 10—C18:0 (Methyl Octadecanoate, 16.4 min); 11—C20:0 (Methyl Arachidate, 20, 20 min); 12—C22:1 (Methyl Erucate, 23.3 min); 13—C22:0 (Methyl Docosanoate, 23.7 min); 14—C24:0 (Methyl Lignocerate, 26.9 min). For the compounds 5 to 10, a 100-fold dilution was performed for the grape seed oil.

When comparing the extraction methodologies, it is noticeable that the percentage of linoleic acid obtained by SFE: C is significantly higher than those obtained by Sox: C and BD: C. For the methodologies with application of pretreatment with ultrasound there was no significant difference between them, however the SFE (with or without pretreatment) may favor the obtaining of linoleic acid, with process time is much shorter than other methodologies, besides of the fact that applied technology offers less aggressive conditions to the compounds of interest that could suffer thermal degradation in conventional processes [26,47]. In addition, Figure 4 shows the fatty acid profile obtained for the method using SFE in the 30:30 condition.

The quality and digestibility of oils are determined by the composition and quantity of unsaturated fatty acids, especially the essential fatty acid content [14,32]. According to the *Codex Alimentarius* Commission [48], the linoleic acid content of grape seed oils averages 57%–78%, higher than in traditionally consumed oils such as corn (50%), soybean (54%), and coconut (46%). The high content of this acid in grape seed oils is particularly interesting as it is the major dietary fatty acid that regulates low density lipoprotein (LDL) metabolism preventing cardiovascular disease [43,47].

Palmitic, stearic and oleic acids were found in higher concentration in Sox extraction. This variation in fatty acid content is possibly related to the extraction methodology, since Sox:C obtained the lowest percentage of polyunsaturated fatty acids, contributing to a proportional increase in SFA and MUFA content [19]. In general, the fatty acid composition was not significantly affected by the extraction method and the ultrasound associated with the extraction methodology contributes to greater extraction of linoleic acid.

### 2.3. Determination of Total Phenolic Content

**Figure 5:**
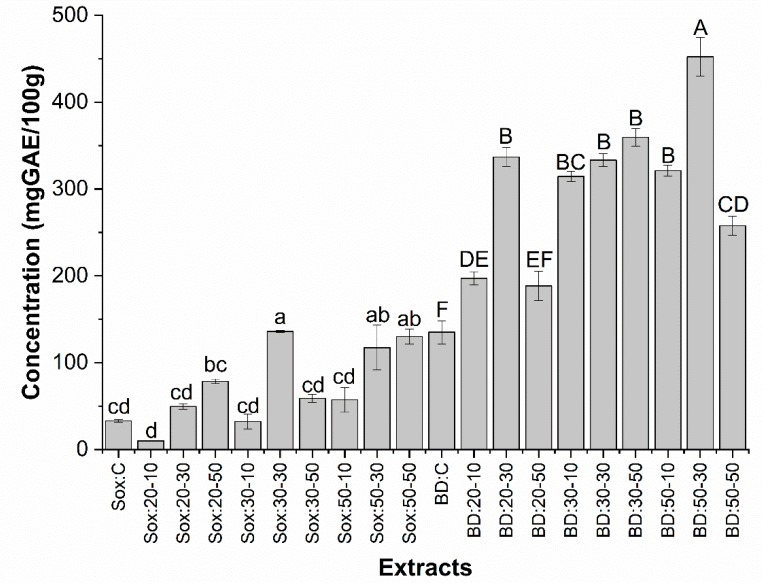
Determination of the total phenolic content of grape seed oils using different treatment conditions (ultrasound) obtained by low pressure methods (Sox and BD). The bars in each column represent the standard error. Samples with the same letter for the same method no significant difference (p > 0.05) by the Tukey test with 95% confidence.

Figure 5 presents the results of the quantification of total phenolic compounds from grape oils obtained by Sox and BD. The content of phenolic compounds ranged from 10.07 ± 0.01 (20-10) to 136.07 ± 1.15 mg GAE/100 g of oil (30-30) for Sox extractions and 135.07 ± 13.27 (C) at 452.40 ± 22.13 mg GAE/100 g oil (50-30) for BD extractions.

The best conditions of phenolic compounds extraction by Sox were: (30:30, 50:30, and 50:50) presenting values of 311%, 255%, and 293% higher when compared to the control without significant differences between them. For the oils obtained by BD it was found that the best condition was 50-30 (452 mg GAE/100g of oil), presenting a 234% higher concentration of phenolics when compared to the control sample. It is therefore possible to state the efficiency of ultrasound pretreatment in increasing the concentration of total phenolics in the oil. This fact could be explained by the influence of ultrasound on the sample hydration and fragmentation process, facilitating the transfer of mass of solutes to the extraction solvent [49].

Otherwise, lower content of phenolic composites submitted when using ultrasound at time greater than 30 min and higher temperatures can probably be attributed to the thermal degradation and polymerization reaction that occurred due to the combination of several phenols, having an effect on analytical quantification [33]. The 30 min ultrasound assisted extraction for phenolic recovery from grape residues has achieved good yields. [50,51]. Khan et al. [52], Virot et al. [53] and González-Centeno et al. [50] showed a 30% increase in the content of phenolic compounds by the effect of ultrasound on orange peel extract; 1.2% in apple pomace and 7% in grape husk extract. In another study Vázquez et al. [54] found an increase (≥5-fold) in the recovery of anthraquinones (phenolic substance) from the rubiaceae stem when ultrasound extraction compared with the Soxhlet method was applied. Similarly, Cai et al. [55] reported that ultrasound extraction increased polyphenol content of purple potatoes by 22% compared to solvent extraction.

**Figure 6:**
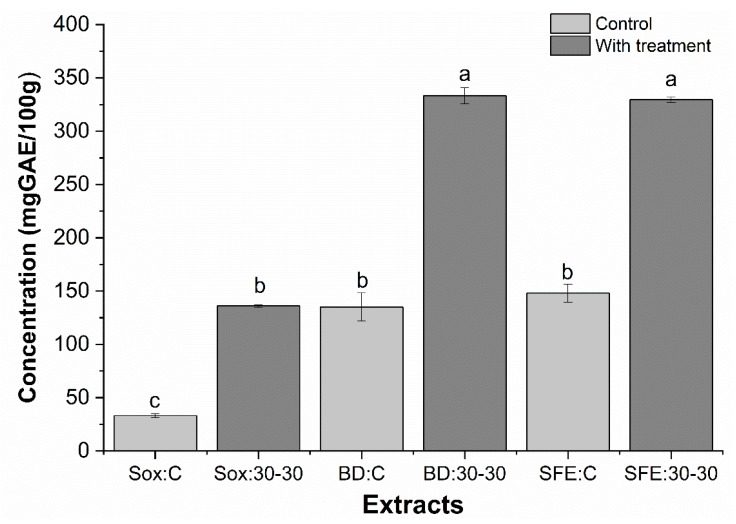
Determination of the total phenolic content of grape oils obtained by low pressure (Sox and BD) and supercritical extraction (SFE) methods, with pretreatment (30:30) and control sample (C). The bars in each column represent the standard error. Samples with the same letter showed no significant difference (p > 0.05) by the Tukey test with 95% confidence.

Figure 6 presents the results for the total phenolic content of grape seed oil obtained by three extraction methods (Sox, BD, and SFE), in the 30:30 pretreatment condition, as well as the respective controls.

Comparing the three applied methods it is observed that the concentration of total phenolic compounds for the pretreated samples, followed the following sequence BD and SFE > Sox and higher when compared with their respective controls. Extraction efficiency may be affected by the chemical nature of phytochemicals, the extraction method used, the particle size of the sample, the solvent used and the presence of interfering substances [56].

The variation in the content of total phenolic compounds between different extraction methods (BD: 30-30; SFE: 30-30 relative to Sox: 30-30) could be explained by the extraction conditions. For BD extraction, chloroform and methanol solvents have nonpolar characteristics, which together with ultrasound benefited the majority extraction of these compounds. On the other hand, SFE uses high pressure (50 MPa) and as solvent CO_2_ operates in an anoxic environment, although it has a low polarity, which may favor the stability of phenolic compounds [26], whereas Sox method can result in the degradation of heat-sensitive compounds (60–70 °C), which are easily hydrolyzed and oxidized, which could contribute to lower antioxidant activity [57,58].

All oil samples with ultrasound pretreatment presented significantly higher phenolic content (p < 0.05) when compared to the control, presenting a yield increase of 311%, 234%, and 184% by Sox, BD and SFE methods, respectively. The positive effect of the ultrasound pretreatment was observed, as well as the negative effect of the high extraction temperatures (Sox) in relation to the phenolic content obtained. The values found for total phenolics with seed pretreatment (136–349 mg GAE/100 g oil) were higher than those found by Mohamed et al. [14]; Lutterodt et al. [19] found 53 mg GAE/g oil and 80 mg GAE/100 g oil, for Pinot Noir and Concoard varieties, extracted by SFE and cold pressing, respectively.

According to Mohamed et al. [14] grape seed oils may be poor in phenolic compounds, a statement not observed in this paper with the application of ultrasound pretreatment, as mentioned above. However, besides the extraction method, the grape variety can influence the concentration of phenolic compounds in the obtained oil, as verified by Baca-Bocanegra et al. [21] who evaluated total phenolic content in grape seed of Syrah variety (60.14 mg GAE/g seed) and Tempranillo variety (57.31 mg GAE/g seed). The higher phenolic concentration of Syrah grape seed can be attributed to the ripening stage, since Syrah grape presents less mature berries than other varieties, which is a relevant factor, since the phenolic extraction capacity in grape seeds decreases during ripening [21]. Other factors such as extraction methodology, which in turn can also profoundly modify the phenolic profile of the extracts; viticultural factors by the pressing operation the contact between monomeric procyanidins such as epicatechin, polyphenoloxidase enzyme, induce its oxidation and polymerization [59]; and geoenvironmental factors [58] may contribute to the phenolic content. Although the results for grape seed oil phenolics were higher than in other studies, the results obtained in grape residue extracts (seed and peel) are superior to those found in oils. Santos et al. [60] and Li et al. [30] found in grape seeds a high content of phenolic compounds ranging from 89.83 to 226 mg GAE/g of seed, much higher results than those found in this study.

### 2.4. Antioxidant Activity and Phenolic Compounds

Table 4 presents the results for antioxidant activity of grape seed oils and phenolic composition of oils obtained by the three extraction methods. IC_50_ values ranged from 54.96 ± 5.68 (SFE: 30-30) to 181.30 ± 77.84 mg/mL (Sox: 30-30) and for the phenolic compounds epicatechin, one of the major compounds in the grape presented values ranging from 1.25 ± 0.03 (SFE: 30-30) to 150.49 ± 5.98 (BD: 30-30). Oils obtained by Sox (Table 4) had higher IC_50_ values, which is defined as the amount of antioxidant required to reduce the initial free radical concentration by 50%, so that the higher the IC_50_ value, the lower antioxidant activity [14]. The IC_50_ values for the sample without Sox and BD pretreatment were lower when compared to the pretreated sample, with no positive effect of ultrasound on the antioxidant activity increase. Samples obtained by SFE showed an increase in antioxidant activity values and, consequently, a reduction in IC_50_ values, justified by the fact that SFE presents high selectivity of the extraction process, as well as a considerable concentration of phenolic compounds that positively correlate with the oil antioxidant [61]. There was no significant difference between the oils obtained with and without SFE seed pretreatment with respect to antioxidant potential. Although the oils studied had significantly different phenolic compounds (p < 0.05) between pretreatment and control samples. This effect was not observed in any of the methodology in the IC_50_ evaluation by the DPPH method. This may be due to the fact that there are other families of bioactive compounds present in oils that may influence the elimination of free radicals such as carotenoids, tocopherols (vitamin E), among others, which may influence antioxidant activity [62].

Fourteen phenolic compounds were evaluated by HPLC in the oil samples obtained by the different methods (Table 4), where significant differences were observed between the phenolic compounds in the different samples. The content of polyphenolic acids ranged from 9.21 mg/kg (Sox: 30-30) to 39.01 mg/kg (SFE: 30-30), with transcinamic acid being the predominant in the samples. Large variations in phenolic acids were observed between the samples. Caffeic acid ranged from 1.62 mg/kg (Sox: 30-30) to 7.16 mg/kg (SFE: 30-30), and p-coumaric acid 0.92 mg/kg (Sox: 30-30). 30) and 11.68 mg/kg (BD: 30-30). Maier et al. [63] studied seven grape varieties (*Vitis vinifera*) and found *p*-coumaric acid (12.3–16.9 mg/kg) and caffeine (9.0–17.4 mg/kg) only in the varieties oils, Lemberger and Carbernet Myths, respectively. Catechin, considered a powerful antioxidant, was detected in all oil samples, regardless of the extraction conditions employed (12.22 ± 0.09 (Sox:C) and 12.32 ± 0.23 (Sox: 30-30), 5.12 ± 0.07 (BD:C) and 15.03 ± 0.76 (BD: 30-30), 3.84 ± 0.25 (SFE:C) and 3.87 ± 0.24 (SFE: 30-30) mg/kg).

The concentration of this compound obtained by BD: 30-30 was approximately 3 times higher (p < 0.05) compared to the BD: C sample, thus demonstrating the efficiency of ultrasound pretreatment in the extraction of this compound. However, the extractions by Sox and SFE showed no significant difference (p > 0.05) between the control sample and the pretreated samples. (-)-Epicatechin was the main flavanol present in oils, with concentrations about 1.5 to 10 times higher than catechin, except for samples obtained by SFE. This result is in agreement with the study conducted by Burin et al. [64] and Rockenbach et al. [65] who found (-)-Epicatechin as the main flavanol present in Cabernet Sauvignon, Merlot and Pinot Noir grapes with concentrations about 2 to 10 times higher than catechin. As for (+)-Catechin, there was a three-fold increase in (-)-Epicatechin content when pretreatment ultrasound was used (BD: 30-30 (150.49 ± 5.98 mg/kg), BD: C (56.68 ± 1.81 mg/kg). For the (+) - Catechin content obtained by Sox and SFE no significant difference was observed between control and pretreated samples, and Sox extraction was significantly higher (p < 0.05) than BD extraction.

Regarding the flavonoid compounds, the concentration of Kaempferol-3-O -glucoside and Quercetin-3-β-D-glucoside was higher when obtained by BD. The flavonoid profile depends on the grape variety, but in general quercetin and its glycosylated derivatives are the predominant compounds [66]. Myricetin was not determined in any of the samples and rutin only in Sox extractions. Radovanović, Radovanović, and Souquet [67] analyzed the phenolic profile of Cabernet Sauvignon Wines from 8 different Balkan regions and showed that the content of phenolic compounds varied depending on the agroclimatic factors and oenological practices of the wine region.

Formononetin was found in all oil samples, with no positive effect of ultrasound on compound extraction. The quantification of formononetin in the oil obtained by SFE:C (108.81 ± 3.86 mg/kg) was 66.4 and 7.6 times higher when compared to BD:C and SOX:C. Formononetin is a specific biomarker of Brazilian red propolis and studies show that this isoflavnoid may have activity against tumor cells [68,69]. No studies were found regarding the identification and quantification of formononetin in grape seed oil.

For stilbenes, the presence of resveratrol was expected because grape seeds are a source of this compound. Thus, it was possible to detect the presence of resveratrol in all samples evaluated, with higher concentration 8.05 ± 0.01 mg/kg (BD: 30-30). The resveratrol content obtained by BD: 30-30 is higher than the content found by Burin et al. [64] 5.6 mg/kg for the Cabernet Sauvignon grape. For BD and Sox extractions, no significant differences were observed between them, as well as they were not influenced by the ultrasound pretreatment. The total amount of resveratrol found in Syrah grape seed oil (BD: 30-30) suggests that it may be considered a grape cultivar with significant resveratrol content, however the extraction methodology may influence its final content [64].

It was verified that the extraction method significantly influenced the extraction of phenolic compounds present in grape seed oil, obtaining the best responses for BD: 30-30. Phenolic compounds are known to have some polarity due to the presence of hydroxyls in their molecules, so their solubility is higher in polar protic media, suggesting that the oil extracted with methanol and chloroform the concentration of some phenolics is higher [70]. It is important to note that supercritical CO_2_ (SFE) extraction was significantly more efficient in obtaining trans-cinnamic acid, caffeic acid and formononetin. Although SFE is not the most efficient method for obtaining larger amounts of phenolic compounds and antioxidant activity of the samples, it is observed that this method provides greater selectivity to obtain p-coumaric acid and formononetin in grape seed oil samples. It is noteworthy that one of the main aspects that should be considered in the SFE is the choice of operating conditions in the extraction process [22,69], as this may provide an additional advantage to conventional methods such as for example clean technology and more selective methodology [69].

The values obtained by HPLC do not correspond to the maximum values obtained with the Folin–Ciocalteau colorimetric method. The total phenolic results obtained by the Folin–Ciocalteau method were much higher than those found by the HPLC. These results can be explained by taking into account that grape seed oils contain other significant compounds such as organic acids, inorganic ions and metal complexes, which may lead to overestimation of total phenolic values [32].

### 2.5. Vitamin E Determination

**Figure 7:**
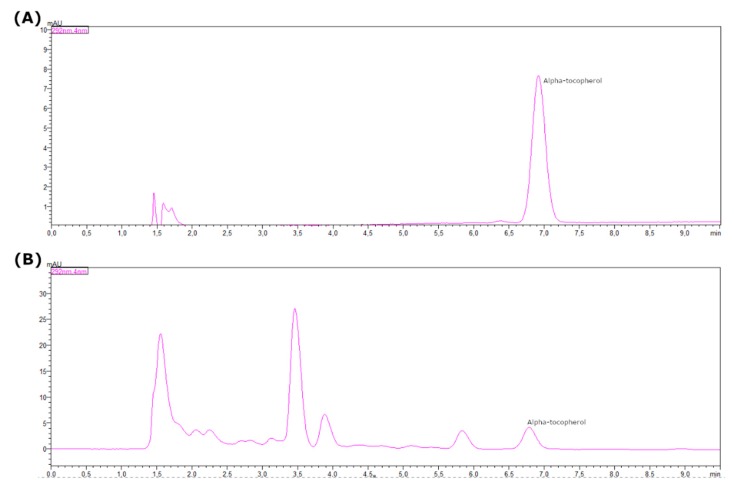
Chromatogram of α-tocopherol standard solution (12.5 mg/L) (**A**) SFE grape seed oil: 30-30 (**B**) obtained by HPLC-DAD at 292 nm.

Figure 7 shows the chromatogram of (A) α-tocopherol standard and (B) grape seed oil. The analysis of oil samples by reverse phase liquid chromatography (C18 RP-HPLC), without saponification or derivatization steps, allowed the quantification of α-tocopherol in grape seed oil with good selectivity and directly.

**Figure 8:**
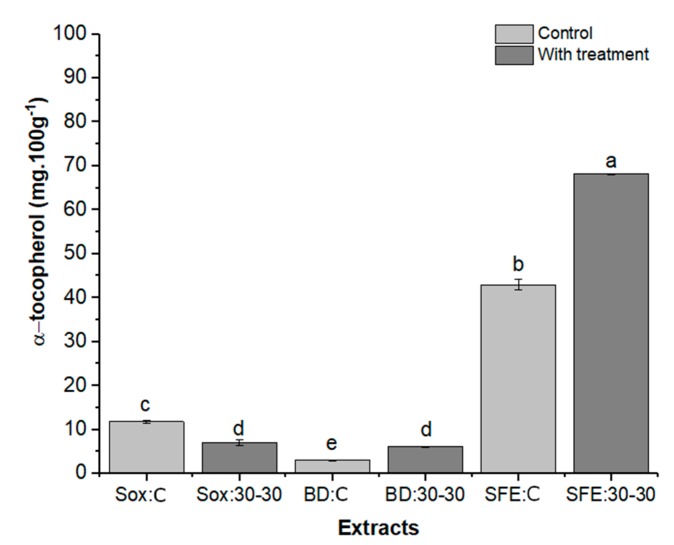
Concentration of α-tocopherol (mg/100 g oil) obtained for grape seed oil submitted to Sox, BD and SFE extractions. Data refer to results for the oil sample without pretreatment (C) and pretreatment (30:30) using ultrasound. The bars in each column represent the standard error. Samples with the same letter show no significant difference (p > 0.05) by the Tukey test with 95% confidence.

Figure 8 presents the results for the yield (mg/100 g) of the oils obtained by the extraction methods (Sox, BD, and SFE) with pretreatment, as well as the respective controls (with-tocopherol (SFE: 30-30), with significant differences between the samples ultrasound treatment). The yield ranged from 3.02 (BD: C) to 68.1 mg/100 g α.

For the samples obtained by SFE and BD it was verified that there was a positive influence of ultrasound, contributing with an increase of approximately 56% and 99%, respectively, in the α-tocopherol content compared to the sample without treatment. The action of ultrasound collapses the plant walls by generating cavitating bubbles, allowing greater release of target compounds [71]. Comparing the extraction methodologies, it was verified that the α-tocopherol content was significantly influenced by the extraction methods and the pretreatment. Sox: 30-30 extraction showed α-tocopherol concentration similar to BD: 30-30, but ultrasound did not have a positive effect for this methodology. The α-tocopherol results obtained from Sox (7.0 and 11.8 mg/100 g oil) and BD (3.0 and 6.0 mg/100g oil) extractions are in accordance with those reported in the literature. as shown by Sabir; Unverb; Karaa [45] who obtained by Sox α-tocopherol contents ranging from 2.7 to 10.6 mg/100 g of oil for 6 grape varieties, Boso et al. [72] studied in 3 grape varieties using cold extraction, and found between 2.9–5.5 mg/100 g of oil. SFE has been shown to be a significantly more efficient (p < 0.05) method for extracting α-tocopherol-rich oil compared to Sox and BD extractions.

Extractions SFE: C and SFE: 30-30 showed 3.6 and 5.7 and 11.2 and 14.2 times, respectively, higher α-tocopherol concentration compared to Sox and BD extractions without and with pretreatment, respectively.

In this study, levels of α-tocopherol 188.2% (SFE:C) and 253.5% (SFE: 30:30) were higher when compared to the best result reported by Mohamed et al. [14], who obtained 17.2 mg α-tocopherol/100 g of grape seed oil (Pinot Noir variety) extracted by SFE (50 MPa, 50 °C) and 120% higher when compared to the maximum value that Beveridge et al. al. [73] observed in oils from 6 grape seed varieties 30.9 mg α-tocopherol/100g (SFE, 37 MPa, 65 °C). These results suggest that several factors influence the amount of α-tocopherol, such as the grape variety used for oil extraction, the variation in supercritical extraction conditions and extraction time [74].

The variation of results for α-tocopherol content in grape seeds, compared to the literature, can be explained by grape varieties, and the methodology employed for the extraction of Vitamin E. According to Lucas et al. [75] SFE may present as a better response for α-tocopherol extraction, due to the possible adjustments in this methodology. Tocopherol is preferentially recovered from the natural matrix in the early stages of extraction, which is normally obtained after the first hour of extraction, and the high affinity of alpha tocopherol to CO_2_ also enables extraction at the beginning of SFE, and is then diluted in oil, extracted over the time of extraction [26,75]. In Soxhlet extraction the long extraction time and temperature used may contribute to decrease vitamin E content due to competitive extraction of other compounds at higher temperature. At higher temperatures, vitamin E extraction should compete with the compound that is easily extracted at the highest extraction temperature such as long chain hydrocarbon and long chain fatty acid [75]. It is also important to note that α-tocopherols are considered highly sensitive to light and air (oxygen) and are recognized as subject to losses by these effects during sample preparation [16,73].

The chemical composition of vegetable oil, and its vitamin E content, is also influenced by genotype, climatic, environmental factors, place of production, growing conditions and variety, environmental factors during harvesting and postharvest storage, and methods [26,74].

### 2.6. Thermal Stability

**Figure 9:**
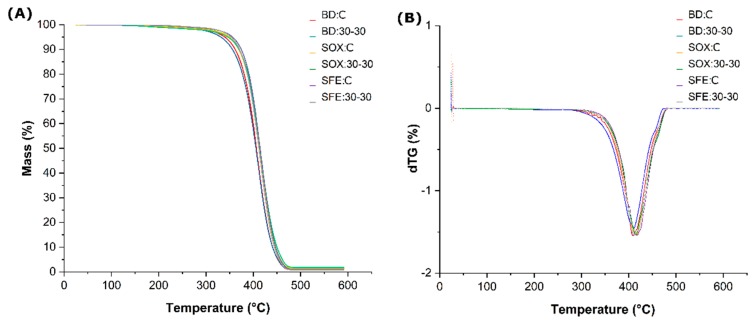
(**A**) Thermogravimetric curve (TGA) (**B**) derivative thermogravimetry (DTG) curves of grape seed oils for supercritical (SFE), Soxhlet (Sox) and Bligh Dyer (BD) extractions from the untreated (Control) and pretreated sample. treatment (30-30).

Grape oil samples obtained by different methods were subjected to thermal analysis which indicated similarity in the decomposition profile (Figure 9). With the analysis of the thermogravimetric curve (TGA) of the evaluated oils it was verified that they presented thermal stability up to 370 °C. The TGA curve (Figure 9A) of the samples reveals that thermal decomposition occurred in a single mass loss step. The only peak on the DTG (derivative thermogravimetry) curve (Figure 9B) indicates that only one degradation reaction occurred between temperatures from 370 to 450 °C.

The thermal decomposition process occurred in the temperature range from 370 to 450 °C for all oils obtained in this study. This temperature is higher than that found by Ontanon et al. [76] and Pereira et al. [32] for sunflower seed oil, and thermally degraded passion fruit seed oil starting at 300 °C. The fatty acid composition of each vegetable oil directly influences its resistance to degradation. Monounsaturated fatty acids are more resistant to oxidation and deterioration compared to unsaturated fatty acids [77]. Although grape seed oil was highly unsaturated, especially due to its linoleic acid content, it presented good thermal stability, which can be attributed to the presence of natural antioxidants such as tocopherols that can contribute to the protection of monounsaturated and polyunsaturated fatty acids. the oxidation. This suggests that grape seed oil can be used in applications that require high temperatures [43,76].

Studies that demonstrate the economic feasibility for scaling up should be performed to assess the behavior of the product obtained. The experimental data found in this study can be useful to support future studies of this type. In addition, the best process conditions found in this work can be used to obtain samples of grape seed oils of different varieties for comparative analysis.

## 3. Material and Methods

### 3.1. Material

Methanol, ethyl acetate, DMSO (dimethyl sulfoxide), isoctane, acetonitrile, acetic acid all HPLC grade obtained from Merck (Darmstadt, Germany) were used. Chloroform, n-hexane (PA) and boron trifluoride (BF_3_) were purchased from Supelco (Supelco Park, PA, USA). 0.45 μm regenerated cellulose membrane filter (SLCR025NS, Millipore Co., Bedford, Massachusetts, USA) was used. The carbon dioxide (CO_2_) used in the extraction was 99.9% pure (White Martins Gases Industriais—São Paulo, Brazil). The standards hydrated rutin (CAS number 207671-50-9), kaempferol (CAS number 520-18-3), formononetin (CAS number 485-72-3), gallic acid (CAS number 149-91-7), quercetin (CAS number 117-39-5), crystalline p-coumaric acid (CAS number 501-98-4), epicatechin (CAS number 490-46-0), caffeic acid (CAS number 331-39-5), catechin (CAS number 7295-85-4), α-tocopherol (CAS number 0010191410), naringenin (CAS number 0067604482), 2,2-diphenyl-1-picrilidrazil (DPPH) (CAS number 1898-66-4), mixture of 14 methyl esters Fatty acid (FAMEs) (Mix C8-C24, CRM 18918 Supelco) were purchased from Sigma-Aldrich Chemical Co and trans-ferulic acid (CAS number 537-98-4) from Fluka.

### 3.2. Obtaining Samples of Grape Seeds

The residue from the production of Syrah wine was kindly provided by the wineries of the São Francisco Submedio Valley (Brazil) (9°23′34″S, 40°30′28″W). The seeds were separated from the skin by hand, and then washed with distilled water, dried in a circulating oven for approximately 20 h (40 °C), vacuum packed and kept at −18 °C until use.

### 3.3. Sample Preparation: Ultrasound Pretreatment Step

The equipment used to perform seed pretreatment was the RMS sonicator—Quimis (Brazil), operated at 50–60 Hz frequency, an ultrasonic input power of 165 W and a usable volume of 2.8 L (internal dimensions: 16.2 × 26.5 × 10 cm). The samples were submitted to different time sonication (10, 30, and 50 min) and temperature (20, 30, and 50 °C) conditions before extraction by the low-pressure methods employed (Sox and BD), as shown in Table 1. Briefly, 50 g of whole grape seed was placed in polyethylene packages, without assistance of any solvent, which were immersed in the ultrasound bath under the predefined conditions (Table 5). After pretreatment with ultrasound, the seeds were ground (Di Grano, Cadence, Brazil) and sieved (Bertel, Tyler, Brazil) to obtain particles with a diameter of less than 0.5 mm, and subsequently subjected to the processing extraction. The best condition obtained for conventional methods (Sox and BD) was employed to obtain the oil by SFE (pretreatment of the sample with 30 min of sonication at 30 °C). For each extraction method used a control sample (C) was obtained corresponding to the non-application of the ultrasound pretreatment. In total, 22 experiments were performed and all extractions were performed in triplicate. SFE was performed after defining the best pretreatment ultrasound condition for conventional techniques (BD and Sox).

After each extraction, the obtained oil yield was calculated according to Equation (1).

(1) $$\mathrm{Yield}\;\left(\%\right)=\frac{m_{\mathrm{oil}}}{m_{\mathrm{seed}}}\times 100$$

### 3.4. Soxhlet Extraction

The conventional Soxhlet extraction was based on the method used by Fiori et al. [35]. Ten grams of sample were placed in the extraction cartridge, and the extraction flask was filled with the extraction solvent (n-hexane). Extraction was performed in a Soxhlet extractor adjusted for 6 h at reflux with 150 mL n-hexane at the boiling temperature of the solvent (60 to 70 °C). Solvent recovery was performed in a rotary evaporator (TE-210, Tecnal, São Paulo-Brazil) with a bath temperature of 40 °C and a rotation speed of 30 rpm. The obtained oils were stored under inert atmosphere (N_2_) conditions to avoid degradation and kept at −18 °C until characterization.

### 3.5. Cold Extraction (Bligh and Dyer)

The extraction procedure was based on the method used by Bligh and Dyer [78]. Ten grams of each sample (6% moisture) were weighed into a conic flask (250 mL), followed by the addition of 10 mL chloroform, 20 mL methanol and 8 mL distilled water. The flask was sealed and shaken for 30 min at 230 rpm on an orbital shaker (MA 420, Marconi, São Paulo-Brazil). Then, 10mL of chloroform and 10mL of 1.5% sodium sulfate were added in the flask mixture which was stirred for 2 min more under the same conditions. The lower chloroform phase containing the lipid fraction was removed and filtered using paper filter with 1.0 g of anhydrous sodium sulfate. Chloroform was evaporated with N_2_ (99.9%, Praxair-Brazil) in a exhaust hood (0216-23, Quimis, São Paulo-Brazil). The obtained oils were stored under inert atmosphere (N_2_) conditions to avoid degradation and kept at −18 °C until characterization.

### 3.6. Supercritical CO_2_ Extraction

After analyzing the results found for the Sox and BD extractions obtained under the different treatment conditions, it was found the best condition (30 min and 30 °C) that was applied to ultrasound treatment and subsequent extraction with supercritical CO_2_ to obtain the grape seed oil (SFE sample: 30-30-Table 1). The Supercritical Fluid Extractor SFT-110 pilot unit (Supercritical Fluid Technologies, Inc.), consisting of a high pressure pump (up to 10,000 psi capacity), extraction cell (100 mL), oven (with heater), static/dynamic valve and restrictor valve, rotameter, totalimeter and CO_2_ cylinder with angler tube (high purity liquid CO_2_, (99.9%, Air Liquide, São Paulo, Brazil). In each experiment (SFE: C and SFE: 30-30) the extraction cell was filled with 60 g of sample. The conditions employed for extraction were: constant temperature 50 °C, pressure 50 MPa, CO_2_ flow 6 g/min totaling approximately 1.5 h extraction [79,80].

### 3.7. Fatty Acid Profile by Gas Chromatography

An aliquot of oil extracted by each extraction method (Sox, BD and SFE) under different conditions was submitted to the transesterification process to prepare the fatty acid methyl esters using the methodology of Joseph and Ackman [81]. The upper phase (isooctane and fatty acid methyl esters) was transferred to 5 mL capacity vials, hermetically sealed and stored at −18 °C under N_2_ atmosphere for further chromatographic analysis. The fatty acid esters were analyzed in a gas chromatograph associated to a mass spectrometer (GCMS-2010 Plus, Shimadzu, São Paulo-Brazil), with a split/splitless injector operating at temperature of 310 °C with capillary column (CP Agilent J&W DB-5ms 30 m × 0.25 mm ID; 0.25 µm film thickness) using helium as carrier gas (White Martins SA, PA, chromatographic grade). The programming performed it was: initial temperature of 70 °C for 2 min, raised to 200 °C at a rate of 30 °C/min and ending at 300 °C to 5 °C/min and a helium flow rate of 1.12 mL/min. The total run time was 33 min. For the mass detector, transfer line temperatures at 280 °C, ion source at 250 °C, and electron impact ionization mode at 70 eV were used. Esterification and injection (1 μL) were performed in triplicate for each sample analyzed. The fatty acid methyl esters were identified and quantified in the Selected Ion Monitoring (SIM) mode and a specific ion was chosen for each analyte. The quantification of the fatty acid methyl esters was performed by normalization of the peak areas in according to [82,83].

### 3.8. Total Phenolic Content

The determination of the total phenolic content was performed according to the method proposed by Capannesi et al. [84]. Briefly, 2.5 g of grape seed oil obtained under different conditions was diluted with 2.5 mL n-hexane and extracted for three times for 10 min and 3000 rpm centrifugation (Sigma, 3-18K, Germany) with methanol: water 80:20 v/v. The obtained sample was added to 2.5 mL Folin–Ciocalteau reagent, 5 mL sodium carbonate (7.5%) in a 50 mL volumetric flask, reaching the final volume with distilled water. Samples were stored for 15 h and spectrophotometric analysis was performed at λ = 765 nm (LAMBDA 25 UV/Vis Systems, Perkin Elmer, Washington-USA). Gallic acid was used as standard (R^2^ = 0.999), and the results were expressed in milligrams of gallic acid equivalents (GAE) per 100 g of sample.

### 3.9. Antioxidant Activity

The in vitro antioxidant activity of the Sox: 30-30, BD: 30-30, SFE: 30-30 samples and their controls (without treatment) was determined using the 2,2-Diphenyl-1-picril-hydrazyl radical (DPPH) according to the adapted methodology of Brand-Williams et al. [85]. DPPH solution (250 μM) was prepared in ethyl acetate, homogenized and kept under light for 1 h to allow formation of the DPPH radical. The samples were weighed and diluted with ethyl acetate to obtain a concentration range of 10 to 110 mg/L. 1 mL of the solution was collected and then 3 mL of the DPPH radical were added. Absorbance reading was performed after 30 min incubation (in the dark) on a UV-Vis spectrophotometer (Lambda 25, Perkin Elmer, USA) at 517 nm. The same procedure was performed with ethyl acetate and considered white. The ability to inhibit free radicals was expressed as the percentage of oxidation inhibition in the radical and calculated according to Equation (2). The IC_50_ value (sample concentration required to inhibit 50% of the DPPH radical) was calculated by line equation based on the concentrations of extracts and their respective percentages of DPPH radical inhibition.

AA (%) = (Aa − Ab) × 100 ÷ AC(2)

where: Aa = absorbance of DPPH solution without addition of sample; Ab = absorbance of DPPH solution mixture and sample; Ac = absorbance of blank solution without DPPH.

### 3.10. High Performance Liquid Chromatography Analysis

Chromatographic analyzes were performed using a Shimadzu High Performance Liquid Chromatograph (HPLC) equipped with a quaternary solvent pumping unit (model LC-20AT), an automatic injector (SIL-20AHT), a diode array detector (SPD- M20A), degasser (DGU-205), column oven (CTO-20A) and a controller interface (CBM-20A). Identification of α-tocopherol was performed using a NUCLEODUR^®^ 100-5 C18 ec column (150 × 4 mm ID; 5 µm particle size) connected to an Agilent ZORBAX Eclipse Plus C18 precolumn (4.6 × 12.5mm).

### 3.11. Phenolic Compounds

Standard stock solutions of phenolic compounds (Sigma-Aldrich and Fluka) were prepared in methanol (HPLC grade) at the following concentrations: gallic acid (540 mg/L); catechin (500 mg/L); caffeic acid (510 mg/L); epicatechin (530 mg/L); p-coumaric acid (550 mg/L); hydrated rutin (505 mg/L) trans-ferulic acid (550 mg/L); myricetin (420 mg/L); resveratrol (560 mg/L); quercetin (545 mg/L); trans-cinnamic acid (520 mg/L); naringenin (540 mg/L); kaempferol (340 mg/L); and formononetin (530 mg/L), except for kaempferol and resveratrol stock solutions which were prepared in DMSO. After optimizing the method (evaluated by injecting a solution containing all the analyte standards) a mixture of 5% acetic acid (solvent A) and acetonitrile (solvent B) was used as the mobile phase. The oven temperature was 40 °C and the mobile phase gradient was 0 to 35 min (0%–92% B); 35 to 40 min (92%–0% B); 40 to 42 min (0% B), with a total run time of 42 min, under a flow rate of 1.0 mL.min^−1^ and an injected volume of 20 µL. The following wavelengths were used for analyzing the phenolic compounds: 280 nm (gallic acid, catechin, epicatechin, trans-cinnamic acid, naringenin), 300 nm (p-coumaric acid, trans-ferulic acid, resveratrol, formononetin, caffeic acid), 320 nm (quercetin), 370 nm (myricetin). Analytical curves were constructed by successive dilutions of an intermediate solution (25 mg/L) containing all the analytes. The following concentration range was used: 0.5 to 15.0 mg/L for phenolic compounds containing seven different concentrations injected in triplicate (R^2^ = 0.999). Moreover, through the parameters of the analytical curves, the limits of detection and quantification of the compounds were obtained. A methanolic extraction was performed using a known concentration of oil according to the method proposed by Capannesi et al. [84]. After obtaining the extracts, they were dissolved in methanol (2000 µg/mL). Quantitative results were expressed as mg phenolic compound per kg oil. All samples and standards were filtered on regenerated cellulose membranes (0.45 µm) prior to injection into the chromatographic system.

### 3.12. Vitamin E

The α-tocopherol standard stock solution was prepared in methanol (HPLC grade) at a concentration of 540 mg/L. After optimizing the method (evaluated by injecting a solution containing the standard), the analyzes were performed in isocratic mode (4% of solvent A and 96% of solvent B), with 5% acetic acid (solvent A) and acetonitrile as mobile phase. (solvent B). The oven temperature was 40 ° C and the total run time 9 min under a flow rate of 1.0 mL/min and an injected volume of 20 µL. The wavelength used in the analyzes was 292 nm. The external standardization method was used to quantify α-tocopherol in grape seed oil. Analytical curves were constructed by successive dilutions of an intermediate solution (25 mg/L) containing the standard. The following concentration range was used: 0.5 to 15.0 mg/L for α-tocopherol, containing eight different concentrations injected in triplicate (R^2^ = 0.999). In addition, through the parameters of the analytical curves, the limits of detection and quantification were obtained.

For the determination of vitamin E concentrations in grape seed oil samples, α-tocopherol extraction was by methanolic extraction using a known oil concentration according to the method proposed by Capannesi et al. [84]. After obtaining the extracts, they were dissolved in methanol (2000 µg/mL). Quantitative results were expressed as mg α-tocopherol per 100 g oil. All samples and standards were filtered on regenerated cellulose membranes (0.45 µm) prior to injection into the chromatographic system.

### 3.13. Thermogravimetric Analysis

Thermogravimetric analyzes of samples BD: 30-30, Sox: 30-30, SFE: 30-30 and their controls (without treatment) were performed on a TGA 50H Shimadzu thermal analyzer. Masses of approximately 11.5 mg, platinum crucible, inert nitrogen atmosphere of 50 mL/min, with heating rate of 10 °C/min, in the temperature range 25 to 600 °C [86]. Results were expressed by TGA curves and by overlapping the DTG (derivative thermogravimetry) curve derived from the first curve.

### 3.14. Statistical Analysis

Results were expressed as mean ± standard deviation (n = 3). Statistical analysis was performed using Statistica (Statsoft Inc., Tusla, USA) version 7.0. Analysis of variance (ANOVA) and Tukey test were used to identify significant differences between the results (p < 0.05).

## 4. Conclusions

It should be noted that the results presented in this work indicated that process ultrasound pretreatment may be a viable means to improve extraction efficiency and obtain phenolic compounds in all extraction methods, specially by SFE, which besides its good efficiency, does not need the use of organic solvents. It is important to highlight, the results identified that the extractions by Sox: 30-30 and SFE 30:30 (13.9%) did not present significant difference among themselves, but they were significantly higher (p < 0.05) than the other samples. Regarding the fatty acid profile, the major one was linoleic acid, and it was observed that the extraction methodology used and the ultrasound application affect the fatty acid profile, confirmed by the increase of 1.80%, 4.05%, and 0.98% major fatty acid (linoleic acid) extracted by Sox: 30-30, BD: 30-30, and SFE: 30-30 respectively, when compared with the control sample. Fatty acid composition was not significantly affected by the extraction method. The oils obtained by Sox showed higher IC_50_ values, therefore they presented the lowest antioxidant activity. The lowest concentrations required for 50% inhibition of DPPH were obtained by SFE, which showed an increase in antioxidant activity values and, consequently, a reduction in antioxidant activity. IC_50_ values Epicatechin was the main flavanol present in grape seed oils, with concentrations about 5 to 10 times higher than catechin, except for the samples obtained by SFE that had lower contents than catechin. For α-tocopherol SFE extraction with and without pretreatment showed up to 14.2-fold higher concentrations of α-tocopherol compared to pretreatment Sox and BD extractions, respectively. In addition, the TGA curve indicated that the oil obtained, for all extraction methods evaluated, has high thermal stability, suffering degradation at temperatures above 370 °C. From the results found in this study, the use of experimental parameters, such as time of application of the pretreatment with ultrasound, as well as temperature, associated with others extraction methods, can benefit the extraction (yield) of several compounds in different types of oils. In addition, it can also contribute to the maintenance of fatty acids of interest in the extracted oil, which on a large scale could result in process savings and higher product quality.

## Figures and Tables

**Table 1:** Comparison of fatty acid profile (% area) of grape seed oil samples using different treatment conditions (ultrasound) by low pressure methods (Sox and BD), the best extraction condition for supercritical fluid (SFE) and their respective untreated controls (C).

*Treatment*	*C8:00*	*C10:0*	*C12:0*	*C14:0*	*C16:1ω7*	*C16:0*	*C18:3ω3*	*C18:2 ω6*	*C18:1 ω9*	*C18:0*	*C20:0*	*C22:1ω9*	*C22:0*	*C24:0*
***Sox:C***	-	-	0.01 ± 0.00	0.07 ± 0.00	0.10 ± 0.00	23.50 ± 0.03	0.16 ± 0.00	57.68 ± 0.46	7.23 ± 0.17	11.04 ± 0.20	0.11 ± 0.43	-	0.033 ± 0.01	0.06 ± 0.02
***Sox: 20-10***	-	-	-	0.04 ± 0.00	0.10 ± 0.00	24.37 ± 0.38	0.18 ± 0.01	56.03 ± 0.35	7.02 ± 0.04	11.47 ± 0.11	0.63 ± 0.00	-	0.09 ± 0.00	0.06 ± 0.01
***Sox: 20-30***	-	-	0.04 ± 0.01	0.31 ± 0.01	0.10 ± 0.00	21.30 ± 0.12	0.17 ± 0.01	59.32 ± 0.20	6.73 ± 0.16	9.60 ± 0.05	0.53 ± 0.01	-	0.94 ± 0.02	0.97 ± 0.15
***Sox: 20-50***	-	-	0.01 ± 0.00	0.09 ± 0.00	0.10 ± 0.00	22.48 ± 0.08	0.18 ± 0.01	60.38 ± 0.19	6.26 ± 0.14	9.50 ± 0.04	0.56 ± 0.02	-	0.19 ± 0.02	0.25 ± 0.01
***Sox: 30-10***	-	-	0.01 ± 0.00	0.10 ± 0.00	0.12 ± 0.01	22.95 ± 0.20	0.20 ± 0.01	59.10 ± 0.18	6.37 ± 0.08	9.65 ± 0.37	0.10 ± 0.02	-	0.21 ± 0.01	0.31 ± 0.01
***Sox: 30-30***	-	-	0.01 ± 0.00	0.07 ± 0.00	0.10 ± 0.00	20.75 ± 0.15	0.21 ± 0.01	61.95 ± 0.22	6.86 ± 0.10	9.20 ± 0.01	0.53 ± 0.00	0.01 ± 0.00	0.13 ± 0.01	0.19 ± 0.02
***Sox: 30-50***	-	-	0.01 ± 0.00	0.03 ± 0.04	0.10 ± 0.01	26.80 ± 0.47	0.14 ± 0.01	53.11 ± 0.48	5.82 ± 0.17	12.72 ± 0.17	0.91 ± 0.02	-	0.17 ± 0.02	0.18 ± 0.05
***Sox: 50-10***	-	-	0.01 ± 0.00	0.06 ± 0.00	0.10 ± 0.01	21.91 ± 0.06	0.16 ± 0.00	59.59 ± 0.02	6.49 ± 0.16	10.68 ± 0.04	0.79 ± 0.07	-	0.10 ± 0.00	0.12 ± 0.04
***Sox: 50-30***	-	-	0.01 ± 0.00	0.06 ± 0.04	0.11 ± 0.01	22.50 ± 0.18	0.17 ± 0.01	59.81 ± 0.20	6.60 ± 0.03	9.86 ± 0.14	0.58 ± 0.01	-	0.14 ± 0.00	0.15 ± 0.05
***Sox: 50-50***	-	-	0.01 ± 0.00	0.06 ± 0.00	0.11 ± 0.00	23.13 ± 0.14	0.16 ± 0.04	57.90 ± 0.03	7.50 ± 0.16	10.39 ± 0.19	0.59 ± 0.01	-	0.08 ± 0.00	0.09 ± 0.01
***BD:C***	-	-	-	-	0.10 ± 0.02	22.15 ± 0.01	0.163 ± 0.01	60.15 ± 0.06	6.64 ± 0.02	10.27 ± 0.02	0.54 ± 0.00	-	-	-
***BD: 20-10***	-	-	0.01 ± 0.00	0.10 ± 0.01	0.10 ± 0.00	20.77 ± 0.22	0.16 ± 0.01	62.17 ± 0.27	6.52 ± 0.02	9.44 ± 0.09	0.50 ± 0.01	-	0.14 ± 0.01	0.10 ± 0.01
***BD: 20-30***	-	-	-	0.02 ± 0.00	0.09 ± 0.00	22.23 ± 0.28	0.18 ± 0.01	60.53 ± 0.38	6.35 ± 0.08	10.00 ± 0.02	0.53 ± 0.01	-	0.04 ± 0.00	0.03 ± 0.00
***BD: 20-50***	-	-	0.01 ± 0.00	0.07 ± 0.00	0.08 ± 0.01	20.22 ± 0.03	0.17 ± 0.00	63.12 ± 0.17	6.46 ± 0.03	9.19 ± 0.11	0.53 ± 0.01	-	0.10 ± 0.00	0.07 ± 0.00
***BD: 30-10***	-	-	0.01 0.00	0.07 ± 0.00	0.10 ± 0.00	20.55 ± 0.22	0.17 ± 0.00	63.08 ± 0.11	6.59 ± 0.23	8.80 ± 0.02	0.46 ± 0.00	-	0.11 ± 0.00	0.07 ± 0.00
***BD 30-30***	-	-	0.01 ± 0.00	0.11 ± 0.05	0.09 ± 0.01	20.40 ± 0.10	0.16 ± 0.00	62.56 ± 0.16	6.39 ± 0.07	9.52 ± 0.06	0.49 ± 0.00	-	0.15 ± 0.00	0.12 ± 0.06
***BD: 30-50***	-	-	0.01 ± 0.00	0.07 ± 0.00	0.07 ± 0.00	27.31 ± 0.19	0.15 ± 0.01	52.39 ± 0.14	5.33 ± 0.01	13.68 ± 0.34	0.73 ± 0.01	-	0.11 ± 0.00	0.15 ± 0.01
***BD: 50-10***	-	-	-	0.05 ± 0.00	0.06 ± 0.00	23.02 ± 0.24	0.20 ± 0.01	59.83 ± 0.11	4.50 ± 0.12	11.52 ± 0.26	0.61 ± 0.02	-	0.09 ± 0.00	0.12 ± 0.01
***BD: 50-30***	-	-	0.01 ± 0.00	0.08 ± 0.00	0.08 ± 0.01	21.73 ± 0.33	0.15 ± 0.01	60.70 ± 0.18	6.16 ± 0.12	10.18 ± 0.08	0.54 ± 0.02	-	0.15 ± 0.00	0.20 ± 0.02
***BD: 50-50***	-	-	0.01 ± 0.00	0.050.00	0.10 ± 0.00	22.86 ± 0.12	0.20 ± 0.03	58.43 ± 0.15	6.98 0.07	10.60 ± 0.11	0.60 ± 0.01	-	0.10 ± 0.00	0.08 ± 0.04
***SFE:C***	-	-	-	0.01 ± 0.00	0.11 ± 0.00	21.04 ± 0.05	0.20 ± 0.01	61.94 ± 0.09	6.34 ± 0.09	10.33 ± 0.03	0.03 ± 0.00	-	0.01 ± 0.01	0.01 ± 0.00
***SFE: 30-30***	-	-	0.00 ± 0.00	0.01 ± 0.00	0.01 ± 0.00	23.02 ± 0.20	0.19 ± 0.01	62.53 ± 0.07	5.65 ± 0.02	8.52 ± 0.12	0.60 ± 0.01	0.02 ± 0.00	-	-

Mean ± standard deviation of analysis (n = 3).

**Table 2:** Sum of fatty acid profile (%) of grape seed oil samples using different treatment conditions (ultrasound) by low pressure methods (Sox and BD), the best extraction condition for supercritical fluid (SFE) and their respective untreated controls (C).

*Treatment*	*Ʃ SFA*	*Ʃ MUFA*	*Ʃ PUFA*
***Sox:C***	34.82 ^c^	7.33 ^ab^	57.84 ^d^
***Sox: 20-10***	36.66 ^b^	7.13 ^bc^	56.21 ^e^
***Sox: 20-30***	33.68 ^cd^	6.83 ^cde^	59.49 ^c^
***Sox: 20-50***	33.09 ^cd^	6.36 ^ef^	60.56 ^b^
***Sox: 30-10***	34.22 ^c^	6.488 ^ef^	59.29 ^c^
***Sox: 30-30***	30.88 ^e^	6.96 ^bcd^	62.16 ^a^
***Sox: 30-50***	40.83 ^a^	5.92 ^g^	53.25 ^f^
***Sox: 50-10***	33.66 ^cd^	6.59 ^def^	59.75 ^bc^
***Sox: 50-30***	33.31 ^d^	6.71 ^cdef^	59.98 ^bc^
***Sox: 50-50***	34.34 ^c^	7.61 ^a^	58.05 ^d^
***BD:C***	32.95 ^D^	6.74 ^B^	60.31 ^B^
***BD: 20-10***	31.05 ^E^	6.62 ^BC^	62.33 ^A^
***BD: 20-30***	32.84 ^D^	6.45 ^BC^	60.71 ^B^
***BD: 20-50***	30.17 ^E^	6.54 ^BC^	63.29 ^A^
***BD: 30-10***	30.06 ^E^	6.68 ^B^	63.253 ^A^
***BD 30-30***	30.80 ^E^	6.48 ^BC^	62.72 ^A^
***BD: 30-50***	42.06 ^A^	5.409 ^D^	52.53 ^D^
***BD: 50-10***	35.41 ^B^	4.56 ^C^	60.03 ^B^
***BD: 50-30***	32.91 ^D^	6.24 ^C^	60.85 ^B^
***BD: 50-50***	34.30 ^C^	7.07 ^A^	58.63 ^c^
***SFE:C***	31.59	5.66	62.75
***SFE: 30-30***	31.51	5.68	62.82

Ʃ SFA, sum of saturated fatty acids; Ʃ MUFA, sum of monounsaturated fatty acids; Ʃ PUFA, sum of polyunsaturated fatty acids; Mean ± standard deviation of analysis (n = 3). Samples with the same lowercase letter (Sox extraction) and the same uppercase letter (BD extraction) on the same column showed no significant difference (p > 0.05) by the Tukey test with 95% confidence.

**Table 3:** Comparison of major fatty acid profile (% area) of grape seed oil samples obtained by three extraction methods (Sox, BD, and SFE) using ultrasound pretreatment (30:30) and their respective untreated controls (C).

Fatty Acid	Sox:C	Sox: 30-30	BD:C	BD:30-30	SFE:C	SFE: 30-30
Palmitic (C16:0)	23.50 ± 0.03 ^a^	20.75 ± 0.15 ^c^	22.14 ± 0.01 ^b^	20.40 ± 0.10 ^c^	21.04 ± 0.05 ^c^	23.02 ± 0.20 ^a^
Stearic (C18:0)	11.04 ± 0.20 ^a^	9.20 ± 0.014 ^c^	10.27 ± 0.02 ^b^	9.52 ± 0.06 ^c^	10.33 ± 0.03 ^b^	8.52 ± 0.12 ^d^
Σ SFA	34.54	29.95	32.41	29.92	31.37	31.34
Palmitoleic (C16:1ω7)	0.10 ± 0.00 ^ab^	0.10 ± 0.00 ^ab^	0.09 ± 0.02 ^bc^	0.09 ± 0.01 ^c^	0.11 ± 0.00 ^a^	0.01 ± 0.00 ^d^
Oleic (C18:1 ω9)	7.23 ± 0.17 ^a^	6.85 ± 0.09 ^b^	6.64 ± 0.02b ^c^	6.38 ± 0.07 ^c^	6.34 ± 0.09 ^c^	5.65 ± 0.02 ^d^
Σ MUFA	7.33	6.95	6.73	6.47	6.45	5.66
α- Linolenic (C18:3ω3)	0.16 ± 0.00 ^c^	0.20 ± 0.01 ^a^	0.16 ± 0.01 ^c^	0.15 ± 0.01 ^c^	0.19 ± 0.01 ^ab^	0.18 ± 0.01 ^b^
Linoleic (C18:2ω6)	57.68 ± 0.46 ^c^	61.95 ± 0.22 ^a^	60.14 ± 0.12 ^b^	62.56 ± 0.16 ^a^	61.94 ± 0.04 ^a^	62.53 ± 0.20 ^a^
Σ PUFA	57.84	62.15	60.30	62.71	62.13	62.71

Mean ± standard deviation of analysis (n = 3). Ʃ SFA, sum of saturated fatty acids; Ʃ MUFA, sum of monounsaturated fatty acids; Ʃ PUFA, sum of polyunsaturated fatty acids. Values that have the same letter, on the same line, do not show significant differences (p > 0.05) by the Tukey test with 95% confidence.

**Table 4:** Determination of antioxidant activity by DPPH (IC_50_) and phenolic compounds (mg/kg) obtained extraction methods (Sox, BD, and SFE) at condition 30:30 and their respective control (C) (without treatment).

Analyses	Method Applied					
	Sox:C	Sox:30-30	BD:C	BD:30-30	SFE:C	SFE:30-30
IC_50_ (mg.mL^−1^)	134.89 ± 13.02 ^ab^	181.30 ± 77.84 ^a^	63.68 ± 2.11 ^bc^	72.36 ± 1.09 ^bc^	56.69 ± 2.74 ^c^	54.96 ± 5.68 ^c^
**Phenolic compounds (mg.Kg^−1^)**						
Kaempferol-3-*O*-glucoside	n.d	2.64 ± 0.03 ^c^	6.32 ± 0.15b ^b^	8.43 ± 0.20 ^a^	n.d	2.83 ± 0.03 ^c^
Rutin	1.87 ± 1.62 ^a^	0.22 ± 0.11 ^b^	n.d	n.d	n.d	n.d
Myricetin	n.d	n.d	n.d	n.d	n.d	n.d
Quercetin-3-β-d-glucoside	4.80 ± 0.02 ^a^	4.91 ± 0.01 ^a^	10.43 ± 9.1 ^a^	7.02 ± 0.32 ^a^	5.17 ± 0.01 ^a^	5.78 ± 0.10 ^a^
**Total Flavonol**	**6.67**	**7.77**	**16.75**	**15.45**	**5.17**	**8.61**
Gallic acid	n.d	n.d	n.d	n.d	n.d	n.d
Caffeic acid	1.81 ± 0.02 ^e^	1.62 ± 0.02 ^f^	6.34 ± 0.03 ^c^	6.98 ± 0.06 ^b^	5.02 ± 0.09 ^d^	7.16 ± 0.05 ^a^
Trans-Ferulic acid	1.32 ± 0.04 ^c^	0.71 ± 0.62 ^c^	3.63 ± 0.04 ^ab^	4.17 ± 0.32 ^a^	4.34 ± 0.04 ^a^	3.35 ± 0.02 ^b^
*p*-Coumaric acid	0.98 ± 0.05 ^e^	0.92 ± 0.02 ^e^	7.30 ± 0.07 ^b^	11.68 ± 0.16 ^a^	3.14 ± 0.14 ^d^	3.42 ± 0.10 ^c^
Trans-Cinnamic Acid	8.48 ± 0,05 ^d^	5.96 ± 0.04 ^d^	18.86 ± 4.20 ^bc^	14.49 ± 0.01 ^c^	20.65 ± 0.12 ^ab^	25.08 ± 0.14 ^a^
**Total phenolics acids**	**12.59**	**9.21**	**36.13**	**37.32**	**33.15**	**39.01**
(–)-Epicatechin	18.8 ± 1.23 ^c^	13.35 ± 0.29 ^c^	56.68 ± 1.8 ^b^	150.49 ± 5.98 ^a^	2.98 ± 0.74 ^d^	1.25 ± 0.03 ^d^
(+)-Catechin	12.22 ± 0.09 ^b^	12.32 ± 0.23 ^b^	5.12 ± 0.07 ^c^	15.03 ± 0.76 ^a^	3.84 ± 0.25 ^d^	3.87 ± 0.24 ^d^
Naringenin	5.05 ± 0.06 ^d^	5.12 ± 0.06 ^d^	17.69 ± 2.45 ^a^	15.99 ± 0.11 ^ab^	13.77 ± 0.14 ^b^	8.14 ± 0.06 ^c^
**Total Flavanol**	**36.07**	**30.79**	**79.49**	**181.51**	**20.59**	**13.26**
Resveratrol	1.04 ± 0.01 ^c^	1.04 ± 0.01 ^c^	3.26 ± 0.62 ^b^	8.05 ± 0.01 ^a^	1.25 ± 0.03 ^c^	1.39 ± 0.80 ^c^
Formononetin	14.25 ± 0.10 ^c^	14.74 ± 0.13 ^c^	1.64 ± 0.04 ^d^	5.98 ± 0.20 ^d^	108.81 ± 3.86 ^a^	34..32 ± 0.46 ^b^

Values with the same letter on the same line showed no significant differences (p > 0.05) in Tukey’s test at 95% confidence. Average of analyses obtained from triplicates (n = 3).

**Table 5:** Experimental parameters employed for the pretreatment of grape seed samples (var. Syrah) with ultrasound (frequency 50–60 Hz), extraction methods investigated for each condition and obtaining controls.

Extraction Methods (Samples Code)	Pretreatment Conditions	
	Temperature of Sonication (°C)	Time of Sonication (min)
**Soxhlet (Hexane)**		
**Sox:C (Control)**	Not applied	Not applied
**Sox:20-10**	20	10
**Sox:20-30**	20	30
**Sox:20-50**	20	50
**Sox:30-10**	30	10
**Sox:30-30**	30	30
**Sox:30-50**	30	50
**Sox:50-10**	50	10
**Sox:50-30**	50	30
**Sox:50-50**	50	50
**Bligh Dyer (Chloroform)**		
**BD:C (Control)**	Not applied	Not applied
**BD:20-10**	20	10
**BD:20-30**	20	30
**BD:20-50**	20	50
**BD:30-10**	30	10
**BD:30-30**	30	30
**BD:30-50**	30	50
**BD:50-10**	50	10
**BD:50-30**	50	30
**BD:50-50**	50	50
Supercritical Fluid* (CO_2_)		
**SFE:C**	Not applied	Not applied
**SFE:30-30**	30	30

BD: Bligh and Dyer; Sox: Soxhlet; SFE: Extraction with supercritical fluid; C (control)-corresponding the control samples. * SFE performed after defining the best pretreatment ultrasound condition for conventional techniques (BD and Sox).

## References

[1] Liang Z., Cheng L., Zhong G.-Y., Liu R.H. (2014). Antioxidant and antiproliferative activities of twenty-four Vitis vinifera grapes. PLoS ONE.

[2] Brenes A., Viveros A., Chamorro S., Arija I. (2016). Use of polyphenol-rich grape by-products in monogastric nutrition. A review. Anim. Feed Sci. Technol..

[3] Cadiz-Gurrea M.D.L.L., Borras-Linares I., Lozano-Sanchez J., Joven J., Fernandez-Arroyo S., Segura-Carretero A. (2017). Cocoa and Grape Seed Byproducts as a Source of Antioxidant and Anti-Inflammatory Proanthocyanidins. Int. J. Mol. Sci..

[4] Lopes de Menezes M., Johann G., Diório A., Pereira N.C., da Silva E.A. (2018). Phenomenological determination of mass transfer parameters of oil extraction from grape biomass waste. J. Clean. Prod..

[5] Rombaut N., Savoire R., Thomasset B., Bélliard T., Castello J., Van Hecke É., Lanoisellé J.-L. (2014). Grape seed oil extraction: Interest of supercritical fluid extraction and gas-assisted mechanical extraction for enhancing polyphenol co-extraction in oil. Comptes Rendus Chim..

[6] Coelho J.P., Filipe R.M., Robalo M.P., Stateva R.P. (2018). Recovering value from organic waste materials: Supercritical fluid extraction of oil from industrial grape seeds. J. Supercrit. Fluids.

[7] García-Lomillo J., González-SanJosé M.L. (2017). Applications of Wine Pomace in the Food Industry: Approaches and Functions. Compr. Rev. Food Sci. Food Saf..

[8] Amorim F.L., de Cerqueira Silva M.B., Cirqueira M.G., Oliveira R.S., Machado B.A.S., Gomes R.G., de Souza C.O., Druzian J.I., de Souza Ferreira E., Umsza-Guez M.A. (2019). Grape peel (Syrah var.) jam as a polyphenol-enriched functional food ingredient. Food Sci. Nutr..

[9] Glampedaki P., Dutschk V. (2014). Stability studies of cosmetic emulsions prepared from natural products such as wine, grape seed oil and mastic resin. Colloids Surf. A Physicochem. Eng. Asp..

[10] Rabrenović B.B., Dimić E.B., Novaković M.M., Tešević V.V., Basić Z.N. (2014). The most important bioactive components of cold pressed oil from different pumpkin (*Cucurbita pepo* L.) seeds. LWT-Food Sci. Technol..

[11] Fernández C.M., Ramos M.J., Pérez Á., Rodríguez J.F. (2010). Production of biodiesel from winery waste: Extraction, refining and transesterification of grape seed oil. Bioresour. Technol..

[12] Manna L., Bugnone C.A., Banchero M. (2015). Valorization of hazelnut, coffee and grape wastes through supercritical fluid extraction of triglycerides and polyphenols. J. Supercrit. Fluids.

[13] Fernandes L., Casal S., Cruz R., Pereira J.A., Ramalhosa E. (2013). Seed oils of ten traditional Portuguese grape varieties with interesting chemical and antioxidant properties. Food Res. Int..

[14] Mohamed B.H., Duba K.S., Fiori L., Abdelgawed H., Tlili I., Tounekti T., Zrig A. (2016). Bioactive compounds and antioxidant activities of different grape (*Vitis vinifera* L.) seed oils extracted by supercritical CO_2_ and organic solvent. LWT.

[15] Lachman J., Hejtmánková A., Táborský J., Kotíková Z., Pivec V., Střalková R., Vollmannová A., Bojňanská T., Dědina M. (2015). Evaluation of oil content and fatty acid composition in the seed of grapevine varieties. LWT-Food Sci. Technol..

[16] Freitas L.D.S., Jacques R.A., Richter M.F., da Silva A.L., Caramao E.B. (2008). Pressurized liquid extraction of vitamin E from Brazilian grape seed oil. J. Chromatogr. A.

[17] Jokić S., Bijuk M., Aladić K., Bilić M., Molnar M. (2016). Optimisation of supercritical CO_2_ extraction of grape seed oil using response surface methodology. Int. J. Food Sci. Technol..

[18] Barba F.J., Zhu Z., Koubaa M., Sant’Ana A.S., Orlien V. (2016). Green alternative methods for the extraction of antioxidant bioactive compounds from winery wastes and by-products: A review. Trends Food Sci. Technol..

[19] Lutterodt H., Slavin M., Whent M., Turner E., Yu L. (2011). (Lucy) Fatty acid composition, oxidative stability, antioxidant and antiproliferative properties of selected cold-pressed grape seed oils and flours. Food Chem..

[20] Da Porto C., Porretto E., Decorti D. (2013). Comparison of ultrasound-assisted extraction with conventional extraction methods of oil and polyphenols from grape (*Vitis vinifera* L.) seeds. Ultrason. Sonochem..

[21] Baca-Bocanegra B., Nogales-Bueno J., Heredia F.J., Hernández-Hierro J.M. (2018). Estimation of Total Phenols, Flavanols and Extractability of Phenolic Compounds in Grape Seeds Using Vibrational Spectroscopy and Chemometric Tools. Sensors.

[22] Duba K., Fiori L. (2019). Supercritical CO_2_ extraction of grape seeds oil: Scale-up and economic analysis. Int. J. Food Sci. Technol..

[23] De Melo M.M.R., Silvestre A.J.D., Silva C.M. (2014). Supercritical fluid extraction of vegetable matrices: Applications, trends and future perspectives of a convincing green technology. J. Supercrit. Fluids.

[24] Machado B.A.S., Pereira C.G., Nunes S.B., Padilha F.F., Umsza-Guez M.A. (2013). Supercritical Fluid Extraction Using CO_2_: Main Applications and Future Perspectives. Sep. Sci. Technol..

[25] Pasquel Reátegui J.L., da Fonseca Machado A.P., Barbero G.F., Rezende C.A., Martínez J. (2014). Extraction of antioxidant compounds from blackberry (*Rubus* sp.) bagasse using supercritical CO_2_ assisted by ultrasound. J. Supercrit. Fluids.

[26] Barrales F.M., Rezende C.A., Martínez J. (2015). Supercritical CO_2_ extraction of passion fruit (*Passiflora edulis* sp.) seed oil assisted by ultrasound. J. Supercrit. Fluids.

[27] Talmaciu A.L., Volf I., Popa V.I. (2015). Supercritical fluids and ultrasound-assisted extractions applied to spruce bark conversion. Environ. Eng. Manag. J..

[28] Moghimi M., Farzaneh V., Bakhshabadi H. (2018). The effect of ultrasound pretreatment on some selected physicochemical properties of black cumin (Nigella Sativa). Nutrire.

[29] Malićanin M., Rac V., Antić V., Antić M., Palade L.M., Kefalas P., Rakić V. (2014). Content of Antioxidants, Antioxidant Capacity and Oxidative Stability of Grape Seed Oil Obtained by Ultra Sound Assisted Extraction. J. Am. Oil Chem. Soc..

[30] Li Y., Skouroumounis G.K., Elsey G.M., Taylor D.K. (2011). Microwave-assistance provides very rapid and efficient extraction of grape seed polyphenols. Food Chem..

[31] Goula A.M. (2013). Ultrasound-assisted extraction of pomegranate seed oil – Kinetic modeling. J. Food Eng..

[32] Pereira M.G., Hamerski F., Andrade E.F., Scheer A.d.P., Corazza M.L. (2017). Assessment of subcritical propane, ultrasound-assisted and Soxhlet extraction of oil from sweet passion fruit (*Passiflora alata Curtis*) seeds. J. Supercrit. Fluids.

[33] Dent M., Dragovic-Uzelac V., Garofulic I.E., Bosiljkov T., Jezek D., Brncic M. (2015). Comparison of conventional and ultrasound-assisted extraction techniques on mass fraction of phenolic compounds from sage (*Salvia officinalis* L.). Chem. Biochem. Eng. Q..

[34] Medina-Torres N., Ayora-Talavera T., Espinosa-Andrews H., Sánchez-Contreras A., Pacheco N. (2017). Ultrasound assisted extraction for the recovery of phenolic compounds from vegetable sources. Agronomy.

[35] Fiori L., Lavelli V., Duba K.S., Sri Harsha P.S.C., Mohamed H.B., Guella G. (2014). Supercritical CO_2_ extraction of oil from seeds of six grape cultivars: Modeling of mass transfer kinetics and evaluation of lipid profiles and tocol contents. J. Supercrit. Fluids.

[36] Samaram S., Mirhosseini H., Tan C.P., Ghazali H.M., Bordbar S., Serjouie A. (2015). Optimisation of ultrasound-assisted extraction of oil from papaya seed by response surface methodology: Oil recovery, radical scavenging antioxidant activity, and oxidation stability. Food Chem..

[37] Araujo G.S., Matos L.J.B.L., Fernandes J.O., Cartaxo S.J.M., Gonçalves L.R.B., Fernandes F.A.N., Farias W.R.L. (2013). Extraction of lipids from microalgae by ultrasound application: Prospection of the optimal extraction method. Ultrason. Sonochem..

[38] Zhang L., Zhou C., Wang B., Yagoub A.E.-G.A., Ma H., Zhang X., Wu M. (2017). Study of ultrasonic cavitation during extraction of the peanut oil at varying frequencies. Ultrason. Sonochem..

[39] Steiner E., Auer A., Becker T., Gastl M. (2012). Comparison of beer quality attributes between beers brewed with 100% barley malt and 100% barley raw material. Sci. Food. Agric..

[40] Hu A., Zhang Z., Zheng J., Wang Y., Chen Q., Liu R., Liu X., Zhang S. (2012). Optimizations and comparison of two supercritical extractions of adlay oil. Innov. Food Sci. Emerg. Technol..

[41] Santos P., Aguiar A.C., Barbero G.F., Rezende C.A., Martinez J. (2015). Supercritical carbon dioxide extraction of capsaicinoids from malagueta pepper (*Capsicum frutescens* L.) assisted by ultrasound. Ultrason. Sonochem..

[42] Markom M., Hasan M., Daud W.R.W., Singh H., Jahim J.M. (2007). Extraction of hydrolysable tannins from Phyllanthus niruri Linn.: Effects of solvents and extraction methods. Sep. Purif. Technol..

[43] Dos Santos Freitas L., Dariva C., Jacques R.A., Caramão E.B. (2013). Effect of experimental parameters in the pressurized liquid extraction of brazilian grape seed oil. Sep. Purif. Technol..

[44] Da Porto C., Natolino A., Decorti D. (2015). Effect of ultrasound pre-treatment of hemp (*Cannabis sativa* L.) seed on supercritical CO_2_ extraction of oil. J. Food Sci. Technol..

[45] Sabir A., Unver A., Kara Z. (2012). The fatty acid and tocopherol constituents of the seed oil extracted from 21 grape varieties (Vitis spp.). J. Sci. Food Agric..

[46] Da Silva A.N.A.C., Jorge N. (2011). Antioxidant Properties of Lentinus Edodes and Agaricus Blazei Extracts. J. Food Qual..

[47] Beres C., Costa G.N.S., Cabezudo I., da Silva-James N.K., Teles A.S.C., Cruz A.P.G., Mellinger-Silva C., Tonon R.V., Cabral L.M.C., Freitas S.P. (2017). Towards integral utilization of grape pomace from winemaking process: A review. Waste Manag..

[48] Alimentarius C. (1999). Codex standard for named vegetable oils. CODEX STAN 210.

[49] Ghafoor K., Choi Y.H., Jeon J.Y., Jo I.H. (2009). Optimization of ultrasound-assisted extraction of phenolic compounds, antioxidants, and anthocyanins from grape (Vitis vinifera) seeds. J. Agric. Food Chem..

[50] González-Centeno M.R., Comas-Serra F., Femenia A., Rosselló C., Simal S. (2015). Effect of power ultrasound application on aqueous extraction of phenolic compounds and antioxidant capacity from grape pomace (*Vitis vinifera* L.): Experimental kinetics and modeling. Ultrason. Sonochem..

[51] Christina D., Kyriakopoulou K., Bimpilas A., Tsimogiannis D., Krokida M. (2015). A comparative study on different extraction techniques to recover red grape pomace polyphenols from vinification byproducts. Ind. Crops Prod..

[52] Khan M.K., Abert-Vian M., Fabiano-Tixier A.-S., Dangles O., Chemat F. (2010). Ultrasound-assisted extraction of polyphenols (flavanone glycosides) from orange (*Citrus sinensis* L.) peel. Food Chem..

[53] Virot M., Tomao V., Le Bourvellec C., Renard C.M.C.G., Chemat F. (2010). Towards the industrial production of antioxidants from food processing by-products with ultrasound-assisted extraction. Ultrason. Sonochem..

[54] Barrera Vázquez M.F., Comini L.R., Martini R.E., Núñez Montoya S.C., Bottini S., Cabrera J.L. (2014). Comparisons between conventional, ultrasound-assisted and microwave-assisted methods for extraction of anthraquinones from Heterophyllaea pustulata Hook f. (Rubiaceae). Ultrason. Sonochem..

[55] Cai Z., Qu Z., Lan Y., Zhao S., Ma X., Wan Q., Jing P., Li P. (2016). Conventional, ultrasound-assisted, and accelerated-solvent extractions of anthocyanins from purple sweet potatoes. Food Chem..

[56] Do Q.D., Angkawijaya A.E., Tran-Nguyen P.L., Huynh L.H., Soetaredjo F.E., Ismadji S., Ju Y.-H. (2014). Effect of extraction solvent on total phenol content, total flavonoid content, and antioxidant activity of Limnophila aromatica. J. Food Drug Anal..

[57] Bimakr M., Rahman R.A., Taip F.S., Adzahan N.M., Sarker M.Z.I., Ganjloo A. (2012). Optimization of ultrasound-assisted extraction of crude oil from winter melon (Benincasa hispida) seed using response surface methodology and evaluation of its antioxidant activity, total phenolic content and fatty acid composition. Molecules.

[58] Hogervorst J.C., Miljić U., Puškaš V. (2017). Extraction of bioactive compounds from grape processing by-products. Handbook of Grape Processing By-Products.

[59] Monrad J.K., Howard L.R., King J.W., Srinivas K., Mauromoustakos A. (2010). Subcritical Solvent Extraction of Procyanidins from Dried Red Grape Pomace. J. Agric. Food Chem..

[60] Santos L.P., Morais D.R., Souza N.E., Cottica S.M., Boroski M., Visentainer J.V. (2011). Phenolic compounds and fatty acids in different parts of Vitis labrusca and V. vinifera grapes. Food Res. Int..

[61] Hasmida M.N., Nur Syukriah A.R., Liza M.S., Mohd Azizi C.Y. (2014). Effect of different extraction techniques on total phenolic content and antioxidant activity of Quercus infectoria galls. Int. Food Res. J..

[62] Pellegrini N., Colombi B., Salvatore S., Brenna O.V., Galaverna G., Del Rio D., Bianchi M., Bennett R., Brighenti F. (2007). Evaluation of antioxidant capacity of some fruit and vegetable foods: Efficiency of extraction of a sequence of solvents. J. Sci. Food Agric..

[63] Maier T., Schieber A., Kammerer D.R., Carle R. (2009). Residues of grape (*Vitis vinifera* L.) seed oil production as a valuable source of phenolic antioxidants. Food Chem..

[64] Burin V.M., Ferreira-Lima N.E., Panceri C.P., Bordignon-Luiz M.T. (2014). Bioactive compounds and antioxidant activity of Vitis vinifera and Vitis labrusca grapes: Evaluation of different extraction methods. Microchem. J..

[65] Rockenbach I.I., Rodrigues E., Gonzaga L.V., Caliari V., Genovese M.I., de Souza SchmidtGonçalvesc A.E., Fett R. (2011). Phenolic compounds content and antioxidant activity in pomace from selected red grapes (*Vitis vinifera* L. and *Vitis labrusca* L.) widely produced in Brazil. Food Chem..

[66] Georgiev V., Ananga A., Tsolova V. (2014). Recent advances and uses of grape flavonoids as nutraceuticals. Nutrients.

[67] Radovanovic B.C., Radovanovic A.N., Souquet J.-M. (2010). Phenolic profile and free radical-scavenging activity of Cabernet Sauvignon wines of different geographical origins from the Balkan region. J. Sci. Food Agric..

[68] Zhang X., Bi L., Ye Y., Chen J. (2014). Formononetin induces apoptosis in PC-3 prostate cancer cells through enhancing the Bax/Bcl-2 ratios and regulating the p38/Akt pathway. Nutr. Cancer.

[69] Machado B.A.S., Silva R.P.D., Barreto G.D.A., Costa S.S., Da Silva D.F., Brandão H.N., Da Rocha J.L.C., Dellagostin O.A., Henriques J.A.P., Umsza-Guez M.A. (2016). Chemical composition and biological activity of extracts obtained by supercritical extraction and ethanolic extraction of brown, green and red propolis derived from different geographic regions in Brazil. PLoS ONE.

[70] Galanakis C.M., Schieber A. (2014). Recovery and utilization of valuable compounds from food processing by-product. Food Res. Int..

[71] Chemat F., Rombaut N., Sicaire A.-G., Meullemiestre A., Fabiano-Tixier A.-S., Abert-Vian M. (2017). Ultrasound assisted extraction of food and natural products. Mechanisms, techniques, combinations, protocols and applications. A review. Ultrason. Sonochem..

[72] Boso S., Gago P., Santiago J.-L., Rodríguez-Canas E., Martínez M.-C. (2018). New monovarietal grape seed oils derived from white grape bagasse generated on an industrial scale at a winemaking plant. LWT.

[73] Beveridge T.H.J., Girard B., Kopp T., Drover J.C.G. (2005). Yield and composition of grape seed oils extracted by supercritical carbon dioxide and petroleum ether: Varietal effects. J. Agric. Food Chem..

[74] Agostini F., Bertussi R.A., Agostini G., Atti Dos Santos A.C., Rossato M., Vanderlinde R. (2012). Supercritical extraction from vinification residues: Fatty acids, alpha-tocopherol, and phenolic compounds in the oil seeds from different varieties of grape. Sci. World J..

[75] De Lucas A., Martinez de la Ossa E., Rincón J., Blanco M.A., Gracia I. (2002). Supercritical fluid extraction of tocopherol concentrates from olive tree leaves. J. Supercrit. Fluids.

[76] Ontañon I., Sanz J., Escudero A., de Marcos S., Ferreira V., Galbán J. (2015). A modified commercial gas chromatograph for the continuous monitoring of the thermal degradation of sunflower oil and off-line solid phase extraction gas–chromatography–mass spectrometry characterization of released volatiles. J. Chromatogr. A.

[77] Moser B.R. (2012). Preparation of fatty acid methyl esters from hazelnut, high-oleic peanut and walnut oils and evaluation as biodiesel. Fuel.

[78] Bligh E.G., Dyer W.J. (1959). A Rapid Method of Total Lipid Extraction and Purification. Can. J. Biochem. Physiol..

[79] Duba K.S., Fiori L. (2015). Supercritical CO_2_ extraction of grape seed oil: Effect of process parameters on the extraction kinetics. J. Supercrit. Fluids.

[80] Machado B.A.S., De Abreu Barreto G., Costa A.S., Costa S.S., Silva R.P.D., Da Silva D.F., Brandao H.N., Da Rocha J.L.C., Nunes S.B., Umsza-Guez M.A. (2015). Determination of parameters for the supercritical extraction of antioxidant compounds from green propolis using carbon dioxide and ethanol as co-solvent. PLoS ONE.

[81] Joseph J.D., Ackman R.G. (1992). Capillary column gas chromatographic method for analysis of encapsulated fish oils and fish oil ethyl esters: Collaborative study. J. AOAC Int..

[82] Balaraman H.B., Rathnasamy S.K. (2020). Kinetics and microwave-assisted extractive transesterification studies of high octane methyl esters (HOME) from karanja and chicken lard oil using protic deep eutectic solvent. Fuel.

[83] Niemi C., Lage S., Gentili F.G. (2019). Comparisons of analysis of fatty acid methyl ester (FAME) of microalgae by chromatographic techniques. Algal Res..

[84] Capannesi C., Palchetti I., Mascini M., Parenti A. (2000). Electrochemical sensor and biosensor for polyphenols detection in olive oils. Food Chem..

[85] Brand-Williams W., Cuvelier M.E., Berset C. (1995). Use of a free radical method to evaluate antioxidant activity. LWT-Food Sci. Technol..

[86] Ferreira C.D., Conceição E.J.L., Machado B.A.V.S., Rios A.O., Druzian J.I., Nunes I.L. (2016). Physicochemical characterization and oxidative stability of microencapsulated crude palm oil by spray drying. Food Bioprocess Technol..

